# Structural Virology: The Key Determinants in Development of Antiviral Therapeutics

**DOI:** 10.3390/v17030417

**Published:** 2025-03-14

**Authors:** Tanuj Handa, Ankita Saha, Aarthi Narayanan, Elsa Ronzier, Pravindra Kumar, Jitin Singla, Shailly Tomar

**Affiliations:** 1 Department of Biosciences and Bioengineering, Indian Institute of Technology Roorkee, Roorkee 247667, India; tanuj@bt.iitr.ac.in (T.H.); ankita_s@bt.iitr.ac.in (A.S.); pravindra.kumar@bt.iitr.ac.in (P.K.); jsingla@bt.iitr.ac.in (J.S.); 2 Department of Biology, College of Science, George Mason University, Fairfax, VA 22030, USA; anaraya1@gmu.edu; 3 Biomedical Research Laboratory, Institute for Biohealth Innovation, George Mason University, Fairfax, VA 22030, USA; eronzier@gmu.edu

**Keywords:** structural virology, antiviral therapeutics, viral proteins, viral replication enzymes, X-ray crystallography, nuclear magnetic resonance (NMR), cryo-EM, emerging and reemerging viruses, rational drug design, bioinformatics

## Abstract

Structural virology has emerged as the foundation for the development of effective antiviral therapeutics. It is pivotal in providing crucial insights into the three-dimensional frame of viruses and viral proteins at atomic-level or near-atomic-level resolution. Structure-based assessment of viral components, including capsids, envelope proteins, replication machinery, and host interaction interfaces, is instrumental in unraveling the multiplex mechanisms of viral infection, replication, and pathogenesis. The structural elucidation of viral enzymes, including proteases, polymerases, and integrases, has been essential in combating viruses like HIV-1 and HIV-2, SARS-CoV-2, and influenza. Techniques including X-ray crystallography, Nuclear Magnetic Resonance spectroscopy, Cryo-electron Microscopy, and Cryo-electron Tomography have revolutionized the field of virology and significantly aided in the discovery of antiviral therapeutics. The ubiquity of chronic viral infections, along with the emergence and reemergence of new viral threats necessitate the development of novel antiviral strategies and agents, while the extensive structural diversity of viruses and their high mutation rates further underscore the critical need for structural analysis of viral proteins to aid antiviral development. This review highlights the significance of structure-based investigations for bridging the gap between structure and function, thus facilitating the development of effective antiviral therapeutics, vaccines, and antibodies for tackling emerging viral threats.

## 1. Introduction

**Figure 1:**
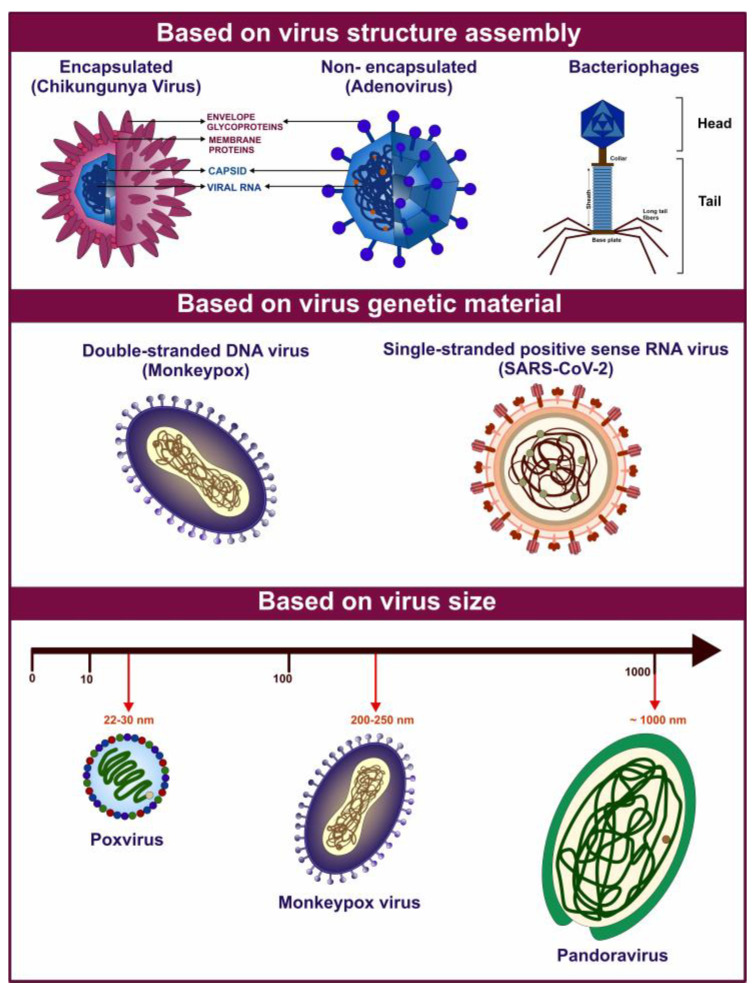
Graphical representation of various viruses exhibiting diverse characteristics based on their structural composition and genetic organization. The figure above highlights examples of various virus types based on their diverse features.

Viruses constitute a diverse group of sub-microscopic infectious agents that are reliant on the host cells’ metabolism to replicate. They lack cellular structures and possess a genome composed of deoxyribonucleic acid (DNA) or ribonucleic acid (RNA), which can be single-stranded or double-stranded. The genome can range from 3000 to over 1,000,000 nucleotides, and virus size can vary from 10 to 1000 nm in diameter [1] (Figure 1). According to the International Committee on Taxonomy of Viruses (ICTV), there are 368 families of viruses as of 2024 [2]. Viruses replicate via a series of complex steps, including initial attachment to the host cell, followed by entry, subsequent uncoating, genome replication, protein synthesis, virion assembly, and release of viral particles. Viral infection can thereby hijack the cellular machinery and disrupt cellular metabolism. These perturbations can manifest as a broad range of pathological outcomes for the host organism and even death [1]. Owing to the wide variety of viruses and the diversity of hosts, including bacteria, blue-green algae, fungi, plants, insects, and vertebrates, viruses pose a threat of infection across the three cellular domains of life- Archaea, Bacteria, and Eukarya.

Archaeal viruses can be broadly divided into archaea-specific viruses and cosmopolitan archaeal viruses, classified into 12 and 5 families, respectively. Most known archaeal viruses have been isolated from extreme environments, from hyperthermophiles or hyperhalophiles. These viruses are known to encode anti-clustered regularly interspaced short palindromic repeats (anti-CRISPR) proteins. It is suggested that the viruses play a significant role in ocean biogeochemical cycling [3,4]. Bacteriophages infect bacteria and exhibit ubiquitous distribution in the environment and diverse genomes. They exhibit lytic or lysogenic life cycles and can facilitate horizontal gene transfer, playing a crucial role in microbial ecology and evolutionary dynamics. Temperate phages can form a mutually beneficial relationship with their host. Phages have played a significant role in developing several molecular biology techniques, including CRISPR-Cas (CRISPR-associated protein) system for genome editing. They have recently been utilized in phage display technology and as phage therapy to combat antimicrobial resistance [5,6]. Amongst eukaryotes, protists, including amoebae, ciliates, and flagellates, can be infected by protist-infecting viruses. Giant viruses (GVs) such as *Mimivirus* belonging to phylum *Nucleocytoviricota* have genome and particle sizes comparable to prokaryotes and small eukaryotes [7,8]. Algal viruses, such as *chloroviruses* and *phaeoviruses* infecting *Chlorella* and brown algae, respectively, influence host evolution via predator-prey selection and genetic exchange, thereby affecting host fitness and microbial community composition. The infections can lead to aquatic “viral shunt”, i.e., alteration of organic matter composition and distribution [9]. Viruses that infect fungi are known as mycoviruses and are classified into 23 families and the genus *Botybirnavirus.* Mycoviruses infecting plant pathogenic fungi are the primary research focus due to their potential to act as biocontrol agents against the host fungi. They are reliant on hyphal anastomosis for intracellular spread and lack an extracellular transmission mechanism, limiting cross-strain spread [10]. Plant viruses pose a significant threat to agriculture and food security and can potentially cause pandemics and epidemics globally [11,12]. They are predominantly RNA viruses transmitted via vectors such as aphids, nematodes, whiteflies, fungi, or mechanical injury. Inside the plant host, they employ plasmodesmata and vasculature to spread internally, exhibiting symptoms such as mosaics, chlorosis, stunting, and wilting. Notable examples include Tobacco mosaic virus (TMV), Potato virus Y (PVY), and Cucumber mosaic virus (CMV) [11,12].

Animal viruses infect an extensive range of hosts, including vertebrates and invertebrates such as insects [1]. They display significant structural and genetic diversity and are categorized into several families. Insect viruses include families such as *Baculoviridae* and *Iridoviridae*, which are known to infect hosts such as *lepidopteran* larvae and other insects [13]. These viruses can be vital for pest management and agriculture. Moreover, arthropods serve as vectors for the transmission of arboviruses, such as members of *Flaviviridae*, *Togaviridae*, and *Nairoviridae***,** to other animals, including humans [14,15,16]. Infection with animal viruses can manifest into numerous disease pathologies, including localized and systemic infection in wild and domestic animals. Members of *Orthomyxoviridae* (e.g., influenza viruses) [17,18] and *Rhabdoviridae* (e.g., rabies virus- RABV) [19] have caused substantial disease burden and mortality. A subset of animal viruses is represented by human viruses, which include notable pathogens, such as members of *Coronaviridae* (e.g., Severe Acute Respiratory Syndrome Coronavirus 2- SARS-CoV-2) [20,21], *Orthomyxoviridae* (e.g., influenza viruses) [17,18], *Herpesviridae* (e.g., herpes simplex virus- HSV) [22,23,24,25], *Papillomaviridae* (e.g., human papillomavirus- HPV) [26,27,28], *Retroviridae* (e.g., human immunodeficiency virus- HIV-1 and HIV-2) [29,30], and *Picornaviridae* (e.g., poliovirus) families [31,32]. Seven identified human oncoviruses, including Epstein-Barr virus (EBV), human T-cell leukemia virus type 1 (HTLV-1), hepatitis B virus (HBV), HPV, hepatitis C virus (HCV), Kaposi’s sarcoma-associated herpesvirus (KSHV) or human herpesvirus 8 (HHV-8), and Merkel cell polyomavirus (MCV or MCPyV) account for causing an estimated 12–15% of cancers globally [33]. Additionally, the zoonotic spillover from animals to humans or reverse zoonosis from humans to animals, potentially facilitated by wildlife farming and trade, is of great concern [34,35]. Examples of zoonoses include rabies and avian influenza. All known human coronaviruses (HCoVs) are believed to have originated in animals, with five of the seven HCoVs originating in bats [36]. Most recently, the Coronavirus disease 2019 (COVID-19) outbreak is believed to have transmitted from bats to humans. Therefore, adopting a One Health approach, considering human, animal, and environmental health, for disease prevention and control is imperative.

Throughout history, viral infections have periodically emerged as epidemics and pandemics, resulting in significant loss of life. It has been estimated that there have been at least 14 influenza pandemics since 1500, including the Russian flu (1889–1893), Spanish flu (1918–1920), Asian flu (1957–1959), Hong Kong flu (1968–1970), and the first influenza pandemic of the 21st century, Swine flu (2009–2010) [37,38]. The ongoing HIV/AIDS pandemic (1981-present) has claimed millions of lives [39,40]. Severe acute respiratory syndrome coronavirus (SARS-CoV) [41], Middle East respiratory syndrome coronavirus (MERS-CoV) [42], and SARS-CoV-2 [20,21,43] are distinct coronaviruses that emerged in 2002, 2012, and 2019, respectively. On 11 March 2020, the World Health Organization (WHO) declared COVID-19, caused by SARS-CoV-2, a pandemic, which raised an alarming situation and caused ~7 million fatalities worldwide [44]. Other significant outbreaks include Smallpox epidemics in the 17th century [45], polio epidemics in the 20th century [46], frequent outbreaks of Ebola [47], Dengue fever [48], yellow fever [49], Zika [50], Measles [51], Chikungunya [52], Japanese encephalitis [53], West Nile fever [54] and rabies [19,55]. As many RNA viruses are emerging and reemerging viruses, they can evolve and reappear in the future with mutations [56,57]. The frequent viral outbreaks and lack of effective treatment and vaccination strategies underscore the urgent need to identify and develop antiviral therapeutics and advanced drug discovery for preparedness against future viral pandemics.

The study of three-dimensional (3D) structures of proteins has been recognized as crucial to expediting drug discovery. It offers insights into the shape of targets, hydrophobic and hydrophilic behaviors of macromolecules, and their interactions with substrates. Structural biology techniques are employed to study the key components of viruses, including structural proteins, replication proteins, and host interaction sites, thereby bridging the gap between viral structure and function, playing a pivotal role in shaping the development of antiviral therapies. The present review provides a comprehensive summary of structure-based investigations in the field of virology that lead to the identification and development of antiviral therapeutics and advanced drug discovery and explores the potential of structural virology in addressing emerging viral threats.

## 2. Exploring the 3D Protein Landscape: Structural Biology Techniques

Structural biology aims to understand the 3D structure of biological macromolecules, including proteins. These techniques have been employed in the study of viruses for close to a century. It not only furthers our understanding of life’s molecular machinery but also enhances our ability to design targeted therapeutic interventions against disease. Researchers can gain valuable insights into biochemical activities and mechanisms by solving protein complex structures, which can be instrumental in drug design, and biotechnology. These methods have helped us visualize the molecular world and expose dynamic and transient stages of proteins [58]. The evolution of structural biology from basic chemical analysis to advanced imaging techniques mirrors scientific inquiry and technology. Chemical degradation and conventional optical microscopy initially provided limited information regarding molecular composition and structure. However, the discovery of X-ray crystallography by the pioneering work of Max von Laue and William Henry Bragg in the early 20th century provided atomic-level resolution and paved the way for structure-guided molecular biology [59,60].

### 2.1. X-Ray Crystallography

X-ray crystallography has been instrumental in antiviral research by enabling the analysis of high-resolution atomic details of crystallized proteins and complexes by interpreting the diffraction patterns [61,62,63]. TMV was the first virus to be crystallized by Wendell Stanley in 1935. It was demonstrated that the infectivity of the virus was retained in crystalline form. He was awarded the Nobel Prize in Chemistry in 1946 [64,65]. The virus structure was described in detail for the first time by Bernal and Fankuchen, who examined TMV suspension via X-ray diffraction [66]. The first atomic resolution structure of a virus was provided in a pioneering study by Harrison et al., revealing an icosahedral arrangement of 180 capsid protein subunits of the Tomato bushy stunt virus (TBSV) at 2.9 Å resolution [67]. Parallelly, Aaron Klug and colleagues determined the structure of the TMV protein disk at a resolution of 2.8 Å [68] and revealed the structure of nucleosome core particle at 7 Å resolution [69]. Aaron Klug was awarded the Nobel Prize in Chemistry in 1982 for his development of crystallographic electron microscopy and his structural elucidation of biologically important nucleic acid-protein complexes [65]. With the development of therapeutics against HIV-1, HIV-2, and HCV, X-ray crystallography became pivotal in antiviral and vaccine research in the late 20th century. Protein crystallization faces challenges such as limited solubility, unresolved protein dynamics, and chemical heterogeneity, complicating structural determination and drug discovery. Additionally, X-ray crystallography offers only static snapshots of molecules, potentially missing important dynamic interactions. The limitations of X-ray crystallography, especially its reliance on crystalline crystals, spurred further advancements [63,70]. While size is not the limitation here, many proteins tend to have flexible regions which makes it difficult to form crystals [71]. Despite these limitations, the impact of X-ray crystallography on fields such as biochemistry, pharmacology, and virology has been profound. The primary advantage of the technique over others is its ability to provide highly detailed atomic resolution structures, essential for understanding the precise molecular mechanics of biological processes. It has enabled the detailed mapping of the interaction sites for drug molecules, providing a foundation for rational drug design and a deeper understanding of fundamental biological processes. Overall, X-ray crystallography remains a vital method in structural biology, complemented by newer techniques that provide insights into the structures of non-crystallizable molecules [63].

### 2.2. Nuclear Magnetic Resonance (NMR)

NMR spectroscopy was developed as a complement to study chemicals, including biomolecules in solution, revealing insights into their conformational flexibility. NMR spectroscopy has evolved from a chemical analysis tool to a fundamental technique in structural biology since the mid-20th century. Advances in technology, higher magnetic field strengths, and computational methods during the 1970s and 1980s allowed NMR to determine the structures of proteins in solution, providing dynamic molecular insights and study of dynamics and interactions within hosts [72]. The technique’s ability to reveal protein dynamics and atomic interactions opened new avenues for the study of protein folding, enzyme activity, and ligand interaction critical to viral pathogenesis and lifecycle, and eventually identifying antivirals to target the viral antigen proteins. NMR serves as a distinctive investigative tool for obtaining atom-resolved information regarding the structural and dynamic characteristics of highly flexible and disordered proteins, such as intrinsically disordered proteins (IDPs). In contrast to the more compact structures of globular protein domains, IDPs significantly influence NMR observables, necessitating the customization of NMR experiments for their study. In this context, ^13^C direct detection NMR has emerged as a valuable instrument for the characterization of IDPs/IDRs at an atomic resolution [73]. NMR is, therefore, uniquely suited for examining physiological states and complex biological processes. However, it is limited by its applicability mainly to smaller proteins (up to about 35 kDa), the requirement of large sample amounts, and extensive time [73]. Its spectral complexity demands high expertise for data interpretation, presenting challenges in high-throughput environments. Despite its limitations, NMR has profoundly impacted structural biology. The conjugation of X-ray crystallography and NMR aids in a deeper understanding of the protein structures [70]. NMR spectroscopy aids antiviral drug discovery by identifying ligand-protein interactions, optimizing drug properties, detecting false positives, and supporting multidisciplinary approaches. NMR spectroscopy can help accelerate the design of antiviral drugs. It has been instrumental in studying the HCV non-structural proteins, including protease, helicase, and polymerase, optimizing drug properties, and validating hits from screening and identifying peptidomimetics against HCV non-structural protein (NS3) serine protease [74].

### 2.3. Transmission Electron Microscopy (TEM)

Electron microscopy is used to visualize the ultrastructure of specimens using focused electron beams. Helmut Ruska made significant contributions to the field of virology by visualizing viruses in the 1930s. In the next decade, he detailed the sub-microscopic structures of various viruses, including poxviruses, TMV, varicella-zoster virus (VZV), and bacteriophages primarily employing TEM [75]. During the 1940s, TEM was employed for the diagnosis of smallpox and chickenpox [76]. In 1959, a negative staining method for high-resolution electron microscopy of viruses was developed [77]. TEM has since been used to understand viral structures, virus-host interaction studies, vaccine development, mutation monitoring, nanomedicine imaging, and diagnostics [78]. The challenges of TEM, such as uneven specimen staining and staining-induced distortions, were overcome when the first successful implementation of cryo-electron microscopy (cryo-EM) was reported.

### 2.4. Cryo-Electron Microscopy (Cryo-EM)

Cryo-EM methodology acquires images of specimens cooled at cryogenic temperatures, aiding in the visualization of proteins, viruses, and complexes in their native state [79]. Its advent revolutionized the field of structural biology as it allowed the study of large protein complexes and fleeting protein states that are hard to crystalize. The technique gained popularity as it facilitates the visualization of biomolecules in their native, hydrated conformations and can achieve near-atomic resolutions comparable to X-ray crystallography without crystallization. With more sensitive detectors and better image processing tools, cryo-EM has become the standard for structural studies of viruses and components such as viral capsids, membrane proteins, and protein complexes [80]. This has provided invaluable insights into viral assembly, infection mechanisms, and interactions with host cells, directly impacting the development of antiviral drugs and vaccines [81]. One of the key advantages of Cryo-EM over other techniques is its ability to study complex and large biomolecular assemblies at near-atomic resolutions, enabling the study of membrane proteins, large protein complexes, and viruses. Additionally, Cryo-EM can capture snapshots of multiple conformational states of a molecule, providing a dynamic perspective on the functional mechanisms. For instance, the structure of RNA polymerase determined using Cryo-EM has advanced the understanding of the dynamic behavior of the enzyme [82]. The technique is not without challenges, including the need for expensive, high-maintenance equipment and the requirement for significant computational resources to process large datasets. Protein size limitations arise as it is almost impossible to obtain images of protein with a size of less than 100 kDa. Limitations can be overcome by targeting the proteins with nanobodies to capture the protein–protein complexes [83]. Moreover, achieving the highest resolutions often necessitates many images and averaging to obtain the 3D structures, which can be time-consuming to collect and analyze. Nevertheless, it enabled unprecedented molecular insights, facilitating structure-guided therapeutic design and driving continual advancements in deciphering intricate biological processes [84].

### 2.5. Small Angle X-Ray Scattering (SAXS)

Building upon the principles of X-ray crystallography, SAXS offers a complementary approach by allowing the study of macromolecules in solution, providing insights into their size, shape, and conformational changes. Unlike X-ray crystallography, which requires the formation of crystals and primarily gives high-resolution static structures, SAXS can analyze samples that are difficult to crystalize and provides low-resolution data on flexible and dynamic assemblies in near-native conditions [85]. SAXS is particularly advantageous for examining large complexes and conducting rapid screenings of samples under various conditions, making it a valuable tool in cases where X-ray crystallography is not feasible. SAXS has been essential in studying IDPs, revealing 3D structures of aggregates, and identifying different stages of protein aggregation due to their flexible domains and smaller size [86]. Conversely, for detailed atomic resolution structures necessary for precise molecular interactions, X-ray crystallography remains the superior technique [87].

### 2.6. Cryo-Electron Tomography (Cryo-ET)

Leaning on the capabilities of cryo-EM, cryo-ET helps study structural biology further by providing detailed 3D visualizations of cells and viruses in their native environment. While cryo-EM offers revolutionary insights into individual proteins and complexes, cryo-ET extends this by allowing scientists to examine the spatial organization and interactions within entire cells or tissues at near-atomic resolutions. In the early 2000s, cryo-ET provided the first visualization of HIV-1 envelope glycoproteins (Env) on the virion surface, revealing their unique tripod-like structure [88]. Subsequent studies have further elucidated Env’s conformational dynamics, aiding in the design of broadly neutralizing antibodies and vaccines [81]. This makes Cryo-ET a superior technique for understanding complex viral infection mechanisms and cellular architecture dynamics, as it captures biological processes in situ without the need for sample sectioning or markers, providing a more comprehensive and realistic view of molecular biology [89].

### 2.7. Emerging Techniques

Together, these techniques complement each other, and ongoing and future advances in technologies such as X-ray Free Electron Laser (XFEL) imaging are broadening the horizons of structural biology as these may disclose new biomolecular behavior, especially in cells and in reaction to inhibitors [90]. Advancements in computational biology have enabled simulations that predict protein folding and dynamics based on known sequences. Techniques such as molecular dynamics (MD) simulations complement experimental data by providing insights into conformational changes over time. During the COVID-19 pandemic, in silico screening and MD simulations of FDA-approved drugs available in the market played a huge role in drug repurposing [43,91,92]. Moreover, AlphaFold 3, the latest iteration of Google DeepMind’s artificial intelligence (AI) tool, offers unparalleled accuracy in predicting 3D protein structures and complex biomolecular assemblies, including protein-nucleic acid and protein-small molecule complexes [93]. This has also helped researchers in modeling protein-based therapeutics against viral infections [93]. In 2024, the Nobel Prize for Chemistry was awarded to David Baker for computational protein design, and to Demis Hassabis and John M. Jumper (Google DeepMind) for protein structure prediction [94].

## 3. Exploring Viral Structural Proteins

Structural proteins are the first to engage with the host receptors during an infection. Structural proteins form the virus’s architecture, comprise a protective outer shell for the genetic material (nucleocapsid), a lipid bilayer containing embedded viroporins (membrane proteins) encasing the capsid, and external proteins facilitating interactions between the virus and host cells (envelope proteins). These proteins are essential for the virus as they depend on these to bind to host receptors, take over host cells, and establish their replication machinery [95]. Adherence to host cells is an essential preliminary phase in the infection process for numerous viruses. Proteins facilitating these interactions have been identified as critical therapeutic targets. For example, The H3 protein in Monkeypox virus (MPXV) is crucial for viral adherence through its interaction with cell-surface heparan sulfate (HS) [96]. Likewise, the E2 protein is crucial for attachment in alphaviruses. Hence, it becomes an essential target for virus entry inhibition [97]. Following attachment, viral infiltration via endocytosis is frequently aided by structural proteins. This process is crucial for virus entry and genome release [98]. In alphaviruses, the E1 protein is pivotal for membrane fusion, facilitating virus entry. As the viral infection progresses, the virus hijacks the host system for the assembly and budding of viruses [99]. Structural proteins are also essential for forming new viral particles within infected cells and their subsequent egress (budding) to infect more cells. For instance, the 6 K protein in alphaviruses, while its function is not entirely elucidated, is believed to contribute to viral budding and enhance membrane permeability [100]. Additionally, numerous viruses possess an internal nucleocapsid core that encases the viral genome. The capsid protein (Cp) constitutes the primary element of the nucleocapsid in many viruses; it engages with the viral RNA and establishes the core structure [101]. Encapsulating the viral genome within the protective protein shell necessitates precise interactions between structural proteins and the viral genetic material. In *Coronaviridae*, the replication and transcription of the viral genome are primarily conducted by the replicase; however, other factors, including viral structural proteins and host proteins, have also been implicated. The coronavirus nucleocapsid protein functions as an RNA chaperone, facilitating template switching in the synthesis of sgRNA [102]. Via attachment, these viruses make critical interactions with the host receptors, which eventually hijack the host cells and develop the virus assembly line. Hence, blocking these interactions of viral structural proteins and host receptors is a crucial step for developing antivirals as entry inhibitors [103]. A structure-guided approach for identifying host receptors that interact with viral proteins, as well as identifying critical residues for viral antigen and host receptor interaction, can act as a therapeutic target covering a range of viruses [104]. Many therapeutics in the form of antivirals and antibodies have been identified against viral structural proteins based on a structure-guided approach and have been in use (Table 1).

### 3.1. Envelope Glycoproteins

Virus envelope glycoproteins are critical structural components that facilitate viral entry into host cells. These proteins play a pivotal role in the infection process by interacting with host cell receptors and enabling viral fusion with the host membrane [105,106]. Envelope glycoproteins can be classified based on their functions into categories such as Spike (S), Envelope (E), or Membrane (M) glycoproteins. S proteins, which protrude from the virus’s surface, are particularly important for attachment to host cells. For example, the S protein of coronaviruses like SARS-CoV-2 is essential for receptor-binding and membrane fusion. Each glycoprotein variant has specific structural features that determine its unique functions. For instance, S proteins often contain receptor-binding domains that enable them to interact with host cell receptors, while some envelope proteins facilitate membrane fusion [102]. Influenza viruses feature hemagglutinin (HA) and neuraminidase (NA) proteins, which regulate the processes of viral entry and exit from host cells [107]. In contrast, HIV-1 and HIV-2 employ the gp160 membrane protein, which splits into gp120, responsible for receptor-binding, and gp41, which aids in membrane fusion [108]. Similarly, SARS-CoV-2 uses its spike (S) proteins to attach to the Angiotensin-converting enzyme 2 (ACE2) receptor in human cells, leading to infection [109]. Viral fusion peptides are essential for viruses to interact and perform endocytosis in the host cell [110]. To further blend with the host system, glycosylation helps enveloped viruses evade immune detection by masking viral epitopes with host-derived glycans, making it challenging for antibodies to identify and neutralize the virus [111]. In other cases, viral envelope proteins are reported to undergo conformational changes that assist in virulence, as observed in SARS-CoV-2 [112] and Dengue virus (DENV) [113].

When viral envelope proteins are detected, the immune system initiates a defense response. While envelope proteins may successfully trigger an immune response, this response is sometimes insufficient to neutralize the virus. For instance, vaccine development for HCV has shown limited effectiveness in generating a robust antibody response [114]. Viruses have developed sophisticated mechanisms to evade these defenses. They suppress interferon (IFN) signaling, inhibit the actions of IFN-stimulated gene products, and disrupt the communication between IFNs and other cellular pathways. These strategies allow viruses to avoid immune detection and maintain their infectivity [115]. Interleukin 10 (IL-10) plays an essential role in supporting Coxsackie B4 virus (CVB4) infection by modulating the immune response to favor viral survival. Interestingly, the antiviral agent Umifenovir has been shown to downregulate IL-10 expression, thereby disrupting the virus’s ability to exploit this pathway. This interaction underscores the importance of IL-10 in viral pathogenesis and highlights the potential of targeting its regulation as an effective strategy for treating CVB4 infections [116] (Table 1).

To escape antiviral treatments, viruses undergo mutations that allow them to target host receptors while avoiding detection by antivirals [117]. Most of these mutations occur in envelope glycoproteins, e.g., the Omicron variant of concern exhibits 37 mutations in its spike protein, which facilitates entry into host cells. Many of these mutations are in two key domains targeted by neutralizing antibodies: the receptor-binding domain (RBD) and the N-terminal domain (NTD). Despite these changes, some therapeutic antibodies, such as 309 (PDB: 7TLY), retain neutralizing activity against Omicron [118]. The conserved region in antigenic site IV in the flank of RBD is conserved due to which the antibody can retain its neutralizing activity [119]. In other cases, envelope proteins can evade immune defenses by binding to antibodies without being neutralized. This phenomenon is observed in the DENV, where antibody binding does not neutralize the virus, leading to a condition called antibody-dependent enhancement (ADE) of infection. ADE occurs during secondary infections and exacerbates disease severity [120,121]. Due to structural similarity between the serotypes, the host responds to viruses with antibodies generated based on previous infection. These antibodies may not bind to neutralizing sites such as fusion loops in DENV or receptor recognition domains which are conserved across virus serotypes [122]. Displaying antigens in a non-infectious manner can help in conferring immunity without compromising the host system with infection. Currently, Dengvaxia is the only vaccine developed against the DENV, and it is effective primarily during secondary infections, as attenuated viruses display the envelope proteins (membrane and envelope glycoproteins) using a different viral vector (yellow fever virus) for the host to generate an immune response but may not lead to infection [123]. Limitations arise as this vaccine cannot be used to immunize prior to primary infection and is only recommended in the case of secondary infection [124]. To combat infections with high mutation rates, such as SARS-CoV-2, targeting highly conserved epitope regions within the antigen is essential. This approach minimizes the risk of immune evasion through mutations [125].

To combat viral infections, entry inhibitors play a critical role by targeting viral envelope proteins and preventing infection [126]. These inhibitors have shown significant efficacy against influenza, one of the most studied viruses in this context. Several potent, Food and Drug Administration (FDA)-approved inhibitors, such as Zanamivir [127], Oseltamivir [128], and Laninamivir octanoate [129], effectively target envelope glycoproteins of influenza virus and block its entry into the host [130] (Table 1). Glycoproteins also have significant potential in vaccine development. When applied appropriately, they can act as antigens and stimulate immune responses, as seen in respiratory syncytial virus (RSV) subunit vaccines for pregnant women and the elderly [131]. Virus-like particles (VLPs) encoding envelope glycoproteins have been effective for multiple purposes, including as diagnostic tools and vaccines [132,133]. With emerging applications, it has been possible to identify antivirals based on entry sites and inhibit virus entry into the host.

### 3.2. Viroporins

Viroporins are small, hydrophobic proteins encoded by viruses. They oligomerize within host cell membranes, forming hydrophilic pores that disrupt cellular processes [134]. Many viruses incorporate membrane proteins into their lipid bilayer envelopes, which are essential for viral entry, assembly, release, and structural integrity. These proteins act as scaffolds, supporting other viral proteins and encapsulating viral DNA [135]. Viroporins facilitate viral budding by interacting with viral proteins and host cell membranes. They also influence membrane fluidity and fusion processes critical for viral entry, although they do not directly bind to receptors like spike or envelope proteins [136].

Viroporins were first identified in 1992 with the discovery of the ion channel activity of the M2 protein of the influenza A virus [137]. Since then, numerous viroporins with diverse structural and functional characteristics have been discovered in various viruses. These proteins are classified into two main groups, class I and class II, based on the number of transmembrane domains they possess. Subclasses are further defined by their position within the membrane [134]. Despite their importance, the study of viroporins as potential targets for antiviral drugs remains challenging. This is due to the lack of reliable 3D structures, difficulties in functional characterization, and the absence of direct, verifiable binding between inhibitors and viroporins [134]. Nevertheless, while the presence of viroporins may not always be critical for viral survival, their absence significantly weakens the virus [138].

Viroporins perform diverse functions across different virus families, reflecting adaptations to specific hosts and biological environments. Membrane proteins contribute to viral envelope stabilization and may also participate in cell signaling and immune evasion. Currently, Amantadine [139], and Rimantadine [140] are the only FDA-approved antivirals targeting the influenza M2 protein. Amantadine binds with the N-terminal of the M2 channel protein for inhibition [141]. It is also reported to have antiviral efficacy against the chikungunya virus ion channel [142], showcasing potential as broad-spectrum viroporins inhibitor. Although Rimantadine has been shown to have antiviral activity against Influenza A, it is reported to show little activity against the influenza B virus and drug-resistant variants arise within a few days after dosage [143]. These drugs elicit antiviral responses, although many other viroporin-targeting therapies remain in pre-clinical and clinical development [144] (Table 1). Advances in cryo-EM have enabled high-resolution imaging of viroporins in their native, membrane-bound conformations. Structural studies reveal the dynamic properties of viroporins, including structural changes during viral entry and membrane fusion [134].

### 3.3. Capsid

Capsids are protective structures in viruses that shield viral genomic material until it enters a host cell [145]. Their primary function is to protect the viral genome from enzymatic degradation by host enzymes and enable its transfer into host cells [146]. Upon entry, the capsid may either disassemble to release the genome for replication or remain intact, allowing transcription within the capsid, depending on the architecture of different virus types [147]. Capsid proteins vary in shape among virus families, containing both major and minor structural proteins. These geometries, studied through models such as Caspar-Klug nomenclature [148] and Alpha shape theory [149], explain assembly patterns in large molecular systems. Computational methods play a vital role in analyzing capsid dynamics, assembly, and interactions with lipid membranes [148]. Capsids are promising antiviral targets due to their essential role in viral infectivity. Disrupting capsid formation or stability can hinder replication [145,150,151,152,153]. Virus-specific non-structural proteins involved in capsid assembly provide opportunities for selective antiviral therapies [154,155]. The nucleocapsid (N) protein of SARS-CoV-2 constitutes a pivotal structural component of the virion, facilitating the encapsulation of the viral RNA into a ribonucleoprotein (RNP) complex and mediating key processes in viral replication and propagation. The C-terminal domain of the N-protein (N-CTD) is indispensable for genome packaging, serving a critical role in the stabilization of the RNP assembly, whereas the RNA-binding site is located in the N-terminal domain (NTD) [156,157]. The first capsid structure identified was that of the TMV [158]. In the 1950s, Rosalind Franklin visualized TMV’s rod-shaped capsid using X-ray crystallography, revealing its protein and RNA organization [159]. Early structural studies employed techniques such as electron microscopy and X-ray crystallography [160,161]. Modern advances, including cryo-EM, have enabled high-resolution imaging of capsids in near-native states, providing insights into structural changes during viral life cycles. Capsid inhibitors are designed to disrupt capsid assembly or disassembly, blocking viral replication. For example, Lenacapavir (marketed as Sunlenca) targets HIV-1 capsids, interfering with multiple replication stages [152]. Immunotherapy strategies also target capsid proteins to elicit immune responses that neutralize viruses. Vaccines often incorporate capsid proteins to stimulate protective immunity [162]. Antivirals based on a structural approach, such as pleconaril, inhibit picornavirus (enteroviruses and rhinoviruses) even with differences in the amino acid sequence of the capsid proteins [163] (Table 1).

## 4. Exploring Viral Non-Structural Proteins

Non-structural proteins are encoded by viral genomes but are not part of the structural components of the virus. These proteins may function to facilitate viral replication or partake in the regulation of replication and assembly. Viral replication enzymes are a subset of viral non-structural proteins encoded by the virus to facilitate the replication and transcription of the viral genome within host cells. Viral replication enzymes, including proteases, polymerases, integrases, and helicases, play crucial roles in synthesizing viral RNA or DNA, enabling genome amplification and the production of new viral progeny. Antiviral drug discovery and repurposing majorly focus on these enzymes (Table 2).

### 4.1. Protease

Virus-encoded proteases catalyze the cleavage of specific peptide bonds in viral polyprotein precursors or in cellular proteins, allowing them to function. Over the years, pre-clinical investigations have focused on these proteases due to their essential role in virus replication. The determination of 3D crystal structures of retroviral proteases began in 1989 with the elucidation of the RSV protease structure, followed by the HIV-1 protease structure [164]. Additionally, the structure of HIV-1 protease was modeled using known eukaryotic aspartic protease structures as templates. The breakthrough in structure-based drug design for viral proteases was enabled when the enzyme and substrate-binding sites of HIV-1 protease (aspartyl protease) were analyzed via X-ray crystallography [165,166,167]. A number of peptide inhibitors (PIs) were designed based on the transition state mimetic concept [168]. The Hoffmann–La Roche drug Saquinavir (Ro 31-8959, Invirase) was the first FDA-approved drug (1995) of HIV-1 protease. The interaction of the drug with the protease was studied by X-ray crystallography [169]. This was followed by Ritonavir (Norvir)’s approval. X-ray structures guided the development, and computational design was incorporated to augment binding and pharmacokinetics [170,171]. Indinavir (Crixivan) was the next HIV protease drug to be approved by the FDA in 1996. Its development was driven by molecular modeling and X-ray crystallographic studies [172,173,174]. Nelfinavir (Viracept) design employed iterative co-crystallographic analyses of protease-bound peptidic inhibitors followed by substitution of parts of the inhibitors with non-peptidic functional groups. It was the first protease inhibitor authorized for pediatric use (1997) [174,175,176]. The other approved HIV antivirals, discovered predominantly employing X-ray diffraction (XRD), include Atazanavir (Reyataz), Darunavir (Prezista), Fosamprenavir (Lexiva), Lopinavir-Ritonavir (Kaletra), and Tipranavir (Aptivus) [62]. In 2013, a joint X-ray/neutron structure HIV-1 protease in complex with now discontinued drug, Amprenavir was determined. The structural data collected by neutron diffraction revealed the precise localization of hydrogen atoms within the active site, which disclosed that some hydrogen bonds may be weaker than previously inferred from non-hydrogen interatomic distances. This insight could prove useful for the development of enhanced protease inhibitors [177] (Table 2). NMR has significantly contributed to the development and optimization of many HIV protease inhibitors. Of these, one notable drug is Ritonavir. Two years post-market launch, certain batches failed to meet dissolution standards. Investigations revealed the presence of an additional crystal Form II other than Form I. Solid-state characterization techniques, NMR, and Infrared spectroscopy (IR) confirmed the existence of two distinct crystalline forms (polymorphs) of ritonavir. However, solution-state analysis demonstrated that both forms dissolve to yield identical molecular structures. Therefore, either form could be employed for manufacture provided complete dissolution [178].

The NS3 protein of HCV comprises two distinct functional domains: an N-terminal serine protease domain and a C-terminal RNA helicase domain. NS4a peptide binds to NS3 and serves as a cofactor for polyprotein maturation. The NS3/4a complex imparts proteolysis of the HCV polyprotein. HCV PIs are designed to inhibit this proteolytic activity [179]. The crystal structure of NS3 was published in 1996, revealing a trypsin-like fold and a structural zinc binding site [180], and the crystal structure of NS3/4a complex revealed NS4A peptide intercalates within a β sheet of the enzyme core [181]. The solution NMR structure of N-terminal protease of NS3 published in 1998 revealed insights into its activation and catalytic mechanism [182]. Initial investigations into the suppression of NS3/4A protease concentrated on the identification and optimization of peptide-based inhibitors [179,183]. Boceprevir and Telaprevir (Table 2) were discovered via structure-guided drug design, both of which belong to the ketoamide class of molecules. They feature a ketoamide group that forms a reversible covalent bond with the enzyme’s catalytic serine residue, thereby inhibiting the enzymatic activity [179]. This was followed by the discovery of several inhibitors via structure-guided drug design. High-resolution crystallographic data served as the basis for discovering small molecule inhibitors (Table 2). In 2012, a highly conserved new allosteric pocket at the HCV protease and helicase domain interface was identified via crystallographic fragment-based screening and proposed as a drug target [184].

The main proteases (Mpro), also termed 3-chymotrypsin-like proteases (3CL pro) and Papain-like protease (PLpro), are cysteine proteases encoded by SARS-CoV-2. Mpro (nsP5) and PLpro (nsP3) co-translationally process pp1a and pp1ab polyproteins into mature non-structural proteins (nsPs), making them attractive drug targets [185,186]. Each protomer of Mpro, a homodimer, consists of three domains (I, II, and III). The substrate-binding pocket is located in the interdomain cleft between domains I and II, where a non-canonical catalytic dyad, Cys145-His41, mediates proteolytic cleavage at 11 distinct sites on pp1a and pp1ab. The substrate-binding sites of SARS-CoV-1 and SARS-CoV-2 Mpro exhibit 100% sequence homology; inhibitors of the former were also screened for the latter [187]. X-ray structures of the unliganded SARS-CoV-2 Mpro and its complex with an α-ketoamide inhibitor were reported, which provided the basis for development of improved inhibitors [188]. These two studies resulted in the development of the inhibitor Nirmatrelvir (PF-07321332) [187]. The FDA-approved Paxlovid (a dual-therapy of Nirmatrelvir and Ritonavir) on 25 May 2023 as an oral antiviral pill [189,190] (Table 2). Ensitrelvir (S-217622), the first oral noncovalent and nonpeptidic SARS-CoV-2 Mpro PI clinical candidate, was discovered via virtual screening and optimization of the hit compound using a structure-based drug design strategy (SBDD). X-ray constructure of the enzyme and PI provided insights into binding and interaction [191] (Table 2). In another study, Cryo-EM structure of polyprotein bound and apo form of Mpro highlighted the flexible nature of the active site [192].

### 4.2. Polymerase

Virus-encoded polymerases are enzymes that catalyze the synthesis of nucleic acid, either DNA or RNA for the replication of viruses, making them key targets for antiviral research [193,194,195]. Different types of polymerases include RNA-Dependent RNA Polymerase (RdRp), RNA-Dependent DNA Polymerase or Reverse Transcriptase (RT), DNA-Dependent DNA Polymerase (DdDp), and DNA-Dependent RNA Polymerase (DdRp) [196].

HSV DNA polymerase (UL30) possesses polymerase activity, intrinsic 3′-5′ exonuclease activity, and ribonuclease (RNase) H activity. The first structure of HSV DNA polymerase was published in 2006. It has five conserved structural domains including an NH2-terminal, 3′-5′ exonuclease, thumb, fingers, and palm domains, and an additional pre-NH2-terminal domain at the N-terminal end [197,198]. Cryo-EM structures published in 2024 revealed the dynamics of the UL30 during DNA synthesis and proof-reading [199]. Viral DNA polymerase cryo-EM structures elucidated how Pol and UL42 bind DNA for processive synthesis, with Pol adopting multiple closed-state conformations in the absence of nucleotides. Structures were not elucidated at the time of approval, but recently drug-bound (Foscarnet and Acyclovir) and drug-resistant mutant analyses indicated that resistance mutations alter conformational dynamics rather than drug binding, clarifying selectivity mechanisms [200].

The mature HIV-1 RT is a heterodimer comprising two subunits: the larger p66 subunit (560 amino acids) and the smaller p51 subunit, derived from the first 440 residues of p66. While the p66 subunit harbors the active polymerase and RNase H domains, the p51 subunit is believed to adopt a structural role with analogous subdomains (fingers, palm, thumb, and connection) differing in relative arrangement to support the heterodimer’s functional integrity. Stavudine, Lamivudine, and Tenofovir disoproxil fumarate are nucleoside reverse transcriptase inhibitors (NRTIs) that bind to the active site of polymerase on activation. These drugs were developed based on the mechanism of action; however, the crystal and cryo-EM structures were elucidated later [201,202] (Table 2). Discovered in the late 1980s, non-nucleoside reverse transcriptase inhibitors (NNRTIs) include five approved anti-HIV drugs: nevirapine, delavirdine, efavirenz, etravirine, and rilpivirine. Dapivirine is approved in several countries. They function as noncompetitive inhibitors by engaging the allosteric site of HIV-1 reverse transcriptase (RT), approximately 15 Å from its catalytic domain. This interaction induces conformational alterations that disrupt the enzyme’s catalytic function, thereby inhibiting viral replication. The hydrophobic binding sites of HIV-1 RT and NNRTIs were identified through compound library screening and structural biology analysis. NNRTIs target HIV-1, while HIV-2’s structural features confer innate resistance [201,202,203,204] (Table 2).

The influenza virus RdRp is a heterotrimeric enzyme composed of PA, PB1, and PB2 subunits. Transcription involves a “cap-snatching” process, wherein nascent capped host RNA transcripts are bound by the PB2 subunit, cleaved by the cap-dependent endonuclease (CEN) of the PA subunit, and utilized as primers by the PB1 subunit for viral mRNA synthesis [205]. The reported cryo-EM structure provides a basis for understanding the enzyme’s activity [206]. FDA-approved baloxavir acid and its prodrug Baloxavir Marboxil, which target the conserved active site of the PA proteins in influenza A and B viruses, were developed by rational drug design leveraging the two-metal pharmacophore framework initially established for Dolutegravir. Recognizing that both HIV integrase and CEN utilize divalent metal ions as cofactors for endonuclease activity, DTG’s metal-chelating scaffold was adapted for CEN inhibition. Crystal structures have been elucidated to study the interaction [205] (Table 1).

SARS-CoV-2’s multi-subunit RdRp complex is composed of a catalytic subunit nsP12 and the accessory subunits, nsP7 and nsP8 [207]. The nsP12 subunit is composed of a nidovirus RdRp-associated nucleotidyltransferase (NiRAN) domain, an interface domain, and a catalytic domain at the C-terminus. These domains collectively mediate key functions, including the catalysis of phosphodiester bond formation between nucleoside triphosphates (NTPs), as well as template binding, entry, primer-template release, and polymerization. The drug remdesivir was approved by FDA in 2020 [208]. While docking and molecular dynamics simulations revealed the dynamic interactions and binding pockets, the cryo-EM structures of SARS-CoV-2 RdRP complex with Remdesivir revealed its incorporation into the nascent RNA strand, stalling elongation and provided a rational template for drug design [209,210,211]. In 2021, the FDA issued an emergency use authorization (EUA) for Molnupiravir, a broad-spectrum ribonucleoside analog, originally developed to treat influenza, and discovered via screening a library of compounds [212,213]. Favipiravir, another inhibitor of influenza RdRP [211] was approved for SARS-CoV-2 treatment in several countries [214]. Cryo-EM structures of the three inhibitors bound to RdRp provide crucial data like the mechanism of enzyme catalysis and base-pairing pattern with inhibitors (Table 2). These drugs are designed to mimic natural nucleotides and incorporate themselves into the growing viral DNA or RNA chain, thereby terminating viral replication.

### 4.3. Integrase

HIV integrase (IN) mediates the insertion of viral DNA into host chromosomal DNA, employing two consecutive reactions named 3′-processing (3-P) and strand transfer (ST). The cleavage of Pol polyprotein by HIV protease leads to the generation of IN, a 32-kDa enzyme composed of three domains: the amino-terminal (NTD), catalytic core (CCD), and carboxy-terminal (CTD). The atomic structures of these domains were resolved using X-ray crystallography or solution NMR spectroscopy [215,216,217]. The IN active site at the CCD domain contains the highly conserved DDE motif, responsible for coordinating two Mg (II) ions. The substantial spatial distance between the enzyme active site in the CCD domain and both the NTD and CTD regions suggests a high degree of multimeric organization within the IN enzyme. Within infected cells, integrase (IN) is predominantly localized in the cytoplasm as a component of the viral pre-integration complex (PIC). The minimal functional subunit of the PIC, the Intasome (INT), catalyzes the integration, a process specific to retroviruses [217]. Combination antiretroviral therapy (cART) comprises four pharmacological classes: (i) NRTIs, (ii) NNRTIs, (iii) PIs, and (iv) integrase strand transfer inhibitors (INSTIs) [217,218]. Since approval, INSTIs have assumed a pivotal role in HIV antiretroviral therapy (ART) due to favorable clinical attributes like high antiviral potency with expedited reductions in HIV RNA levels, absence of significant drug–drug interactions, and cross-resistance to other drugs [219].

All INSTIs share structural similarity to experimental compound 1-(5-chloroindol-3-yl)-3-hydroxy-3-(2H-tetrazol-5-yl)propenone (5-CITEP) characterized by the pivotal diketoacid (DKA) moiety. It represents the first integrase inhibitor successfully co-crystallized with the CCD, with electron density confirming interactions with residues D64, D116, and E152, as well as additional contacts involved in host DNA docking [217,220,221]. Further studies revealed that PICs are predominantly assembled via coordination with two Mg(II) ions during viral DNA integration, one of these metal ions is coordinated by residues D64 and D116, whereas the other is coordinated by residues D116 and E152. DKA-containing compounds maintain antiviral activity provided their divalent metal ion chelation capacity is retained. Raltegravir and Elvitegravir are first-generation INSTIs. The widespread use of Raltegravir since its approval in 2007 resulted in the rapid emergence of viral variants harboring mutations within the IN-CCD domain [217]. Second-generation INSTIs include Dolutegravir [222], Bictegravir, and Cabotegravir. Elucidation of crystal structure revealed enhanced structural flexibility of Dolutegravir improved the drug’s integration into the enzyme active site, increasing efficacy against mutations triggered by first-generation INSTIs [217,221] (Table 2). Since 2018, WHO has recommended the use of Dolutegravir as the preferred first- and second-line HIV treatment [223]. Bictegravir and Cabotegravir exhibited superior genetic barriers to resistance [217]. Cryo-EM structures of second-generation INSTIs bound to INTs and drug-resistant INTs further revealed mechanisms of inhibition and resistance and may aid in the development of better drugs [224,225].

X-ray and cryo-EM structures demonstrated that allosteric IN inhibitors (ALLINIs) disrupt the catalytic activity of the enzyme by binding to allosteric sites [217,226]. Lens Epithelium-Derived Growth Factor/p75 (LEDGF/p75) is a cellular factor that enhances host DNA interaction with functionally active PICs by binding to the integrase binding domain (IBD) at the C-terminus IN, via coordination with residues D366, V408, I365, and F406. LEDGINs (coined after Lens epithelium-derived growth factor/p75 cofactor binding pocket on IN) target this interaction and affect IN multimerization and catalytic activity. These can be developed via rational drug design and show no cross-resistance with INSTIs [217,218,227]. Multimerization-selective integrase inhibitors (MINIs) act by promoting aberrant IN multimerization. IN-RT RNase H inhibitors and INI-LEDGF/p75-IN interaction disruptors are examples of dual-acting inhibitors that simultaneously act on different and/or multiple targets in an additive or synergistic manner to confer antiviral effect [217].

### 4.4. Thymidine Kinase (TK)

Thymidine kinase (TK) genes of alpha-herpesviruses are virulence-related genes encoding key kinases in the nucleoside salvage pathway. The HSV-1 TK enzyme phosphorylates four nucleosides and various nucleoside analogs. Hence, this enzyme is a pivotal target in antiviral therapeutic strategies [228]. The crystallographic structures of HSV-1 TK bound to its endogenous substrate deoxythymidine (dT) and the guanosine analog Ganciclovir were elucidated and published in 1995 [229,230]. Three 5-substituted 2′-deoxyuridine analogs (Idoxuridine, Trifluridine, and Brivudine [BVDU]) have been approved as antiviral drugs. These analogs mimic natural substrates, facilitating phosphorylation. These phosphorylated antiviral agents interfere with viral DNA replication (Table 2). Acyclic guanosine analogs like Acyclovir and Penciclovir are phosphorylated by TK to monophosphate form. Cellular enzymes convert the compound to its active triphosphate form, which is then incorporated into viral DNA, halting replication [230] (Table 2). Mutations in TK gene can result in resistance to these inhibitors [231].

### 4.5. Methyltransferase (MTase)

In viral systems, MTases facilitate the synthesis of the 5′ cap-0 structure, thereby enhancing the virus’s ability to evade the host’s innate immune defenses [232]. nsP1 of alphaviruses exhibits S-adenosyl-l-methionine (SAM)-dependent methyltransferase (MTase) and m7GTP transferase (GTase) activities that are membrane-binding dependent and are essential for viral capping [233]. Solution NMR and cryo-EM structures provide insights into the membrane-binding and enzyme capping mechanism [234,235]. Berbamine hydrochloride (BH), ABT199/venetoclax (ABT), ponatinib (PT), and selective estrogen receptor modulators (SERMs) are reported to inhibit nsP1 of Chikungunya Virus (CHIKV) and Sindbis Virus (SINV) [236,237,238].

The plus-strand RNA genome of flaviviruses is capped with a 5′ terminal Cap 1 structure (m7GpppAmG). The flaviviruses encode one methyltransferase, located at the N-terminal portion of the NS5 protein, responsible for catalyzing both guanine N-7 and ribose 2′-OH methylations [239]. X-ray structures aided in the elucidation of the catalytic site and drug-binding pockets [239,240,241,242,243]. Ribavirin, aurintricarboxylic acid, sinefungin, S-adenosyl homocysteine (SAH), Compound 10, herbacetin (HC), and caffeic acid phenethyl ester (CAPE) are reported as DENV MTase inhibitors [244,245,246,247]

SARS-CoV-2 MTases, nsp10/nsp16 and nsp14, facilitate viral RNA capping. Nsp14 catalyzes the 7-methylation of the 5′-cap guanosine, while nsp16 with nsp10, mediates the 2′-O-methylation of the ribose, thereby finalizing the cap structure. X-ray and cryo-EM structures were solved for both enzymes [248]. Several adenosine-like inhibitors and non-nucleoside inhibitors have been reported against nsp14 and nsp16 [249].

### 4.6. Helicases

Virus-encoded helicases are exploited by certain viruses to unwind DNA or RNA duplexes, a process driven by the hydrolysis of ATP [250]. Prominent examples include a domain of HCV-NS3 [251], vaccinia nucleoside triphosphate phosphohydrolase-II (NPH-II) [252], SARS-CoV-2 Nsp13 helicase [253], Simian virus 40 (SV40) TAg protein [254], HPV E1 protein [255], DENV NS3 helicase [256], MPXV E5 helicase [257], and N-terminal helicase of CHIKV nsP2 [258]. Inhibitors are reported to target the helicase-mediated ATP hydrolysis and nucleic acid binding [259]. X-ray [256,260,261,262,263,264,265], cryo-EM [257,266], NMR [267], and structure prediction [266] data have been reported for several viruses. Helicase enzymes are essential during multiple stages of genome replication, rendering them compelling antiviral targets, particularly given the extensive structural data available. Despite significant efforts to develop helicase-targeting antivirals, clinical advancement has been hindered by challenges related to cytotoxicity, bioavailability, and pharmacokinetics [259,268]. As of now, no virus-encoded helicase inhibitors have received FDA approval.

## 5. Host-Targeted Antivirals

**Figure 2:**
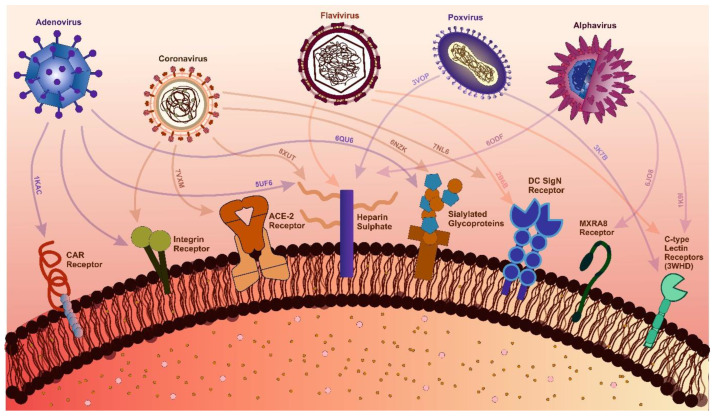
Interactions Between Viral Families and Host Receptors with Structural Insights. The figure highlights key interactions between viral families and host receptors critical for viral attachment and entry. For adenoviruses, the Coxsackievirus, and Adenovirus Receptor (CAR) (PDB: 1KAC), heparan sulfate (PDB: 5UF6), and sialylated glycoproteins (PDB: 6QU6) are key receptors. Coronavirus interactions include ACE-2 (PDB: 7VXM), integrins, heparan sulfate (PDB: 8XUT), sialic acid (PDB: 6NK), and the Dendritic Cell-Specific Intercellular adhesion molecule-3-Grabbing Non-integrin (DC-SIGN) receptor (PDB: 7NL6) [284,285]. Alphaviruses engage with heparan sulfate (PDB: 6ODF), C-type lectin receptors (CLRs) (PDB: 1K9I), and Matrix remodeling-associated protein 8 (MXRA8) (PDB: 6JO8) [99]. Similarly, flaviviruses interact with heparan sulfate, DC-SIGN (PDB: 2B6B), and C-type lectin receptors [286,287]. Poxviruses target heparan sulfate (PDB: 3VOP) and C-type lectin receptors (PDB: 3K7B) [288]. PDB identifiers highlight viral protein-host receptor complexes, offering molecular-level insights into receptor diversity and viral entry mechanisms, supported by recent structural and experimental studies.

Protein–protein interactions (PPIs) are fundamental to any virus infection. A detailed understanding of protein interactions is essential for understanding viral pathogenesis. Many host proteins act as receptors for viruses, mediating attachment and entry (Figure 2). C-C chemokine receptor type 5 (CCR5) and C-X-C chemokine receptor type 4 (CXCR4) act as co-receptors for the entry of HIV-1. Antagonists of these co-receptors are being utilized as antiviral strategies. Drug candidates like Aplaviroc, Cenicriviroc and Vicriviroc have shown efficacy in inhibiting HIV-1 replication. Available structural data for CCR5 and CXCR4 can prove to be valuable for improving inhibitor design and therapeutic potential [269,270,271]. Structure of the CCR5 chemokine receptor with FDA-approved inhibitor, Maraviroc has been reported [269]. Ibalizumab, a cluster of differentiation 4 (CD4)-directed post-attachment inhibitor was the first monoclonal antibody to be approved for the treatment of HIV-1 infection [272,273].

**Figure 3:**
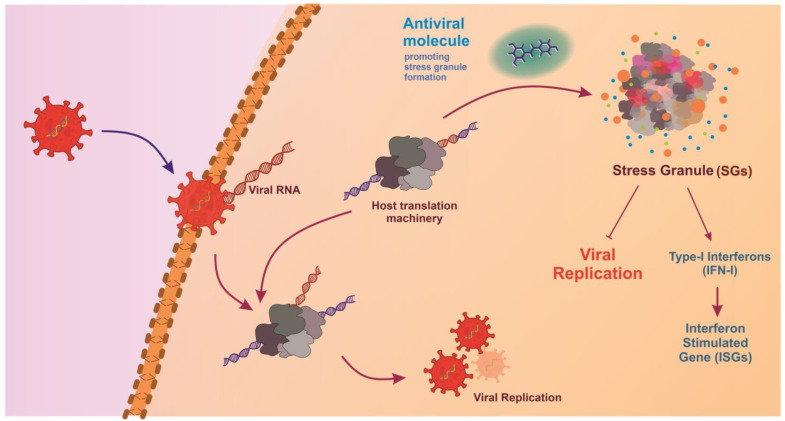
Stress granule formation in response to viral infection. Viruses hijack host translational machinery to promote viral replication. Antiviral molecules promote stress granule formation, leading to the production of Type I Interferons (IFN-I) and activation of Interferon-Stimulated Genes (ISGs), directly inhibiting viral replication, thereby creating an antiviral environment that restricts viral survival and propagation.

Additionally, glycosylation, autophagy, actin polymerization, fatty acid biosynthesis, programmed ribosomal frameshifting (PRF), and proteolytic cleavage are some of the host-mediated metabolic processes that can be targeted for antiviral development. Key pathways that can be targeted include host lipid pathway, host glycolytic pathways, host ubiquitination pathways, polyamine metabolic pathway, host nucleoside synthesis pathway, cytokine signaling, and inflammatory pathways, and stress granule (SGs) machinery (Figure 3) [274,275]. SGs are assemblies of stalled mRNA and proteins that form in response to cellular stress, such as viral infection. Viral proteins like N-protein of SARS-CoV-2 and nsP3 of CHIKV in virus-infected cells recruit SG proteins G3BP1 (Ras GTPase-activating protein SH3-domain-binding protein 1) and G3BP2. This interaction suppresses SG formation, enhancing virus replication and assembly of new virions. Therefore, antivirals targeting the host protein G3BP can aid in SG-mediated antiviral response [276,277]. Viruses establish virus–host protein interactions to exploit cellular machinery essential for critical stages such as entry, genome replication, translation, assembly, and release [278,279,280]. Increased resistance to antiviral agents and the need for broad-spectrum antiviral therapeutics have steered efforts to target proviral host factors and cellular mechanisms [274,275,281,282,283].

Viral infection can lead to the activation of various innate immune responses, with interferons (IFNs) playing a central role. IFNs, particularly IFN-α, have been utilized in antiviral therapies for infections like hepatitis B and C, with ongoing efforts to enhance therapeutic efficacy. Pegylated interferon afla 2b (PegIFNα-2b), interferon alfacon 1 (CIFN), pegylated interferon alfa 2b + ribavirin (PegIFNα-2b+RBV), pegylated interferon afla 2a (PegIFN-α2a) are some of the interferon therapeutics that are approved against HCV infections. Podofilox (PDX) is an antimitotic drug that interrupts cell division, approved for HPV-related diseases. Imiquimod (IQM) stimulates cytokines and sinecatechins (SINE) is an immunomodulatory drug approved against HPV-related diseases [62].

Cyclophilins (Cyps), are key cellular factors playing a role in transcription regulation, immune response, protein secretion, and mitochondrial function. Cyclophilin A (CypA), a mediator of cyclosporin A’s (CsA) immunosuppressive effects, also supports the replication of multiple viruses, including HIV-1, HCV, and influenza. Interaction of CypA with viral proteins, facilitates virus replication, as seen with HIV-1, HCV, influenza virus, HCV, VSV, vaccinia virus, SARS-CoV, rotavirus (RV), and HPV. Cyclophilin inhibitors, such as alisporivir and NIM811, are reported to display potent antiviral activity against HIV and HCV [259].

## 6. Rational Drug Design

Viral outbreaks, such as COVID-19 and Monkeypox, highlight the challenges posed by viral mutations that enhance immune escape and virulence, potentially leading to pandemics. Predicting the next mutation remains difficult [289]. Therefore, global preparedness is essential to counter future pandemics [290]. The pursuit of advanced, structure-guided treatments through AI-driven technologies is crucial for addressing viral mutations that could reduce therapeutic efficacy [291]. For instance, after the COVID-19 outbreak, it became evident that a strategic pipeline is required to identify antigenic sites, design non-cytotoxic ligands, and enable mass production and distribution of drugs [292]. Similarly, the Monkeypox outbreak reinforced the need to identify viral targets and develop specific drugs against them [293].

**Figure 4:**
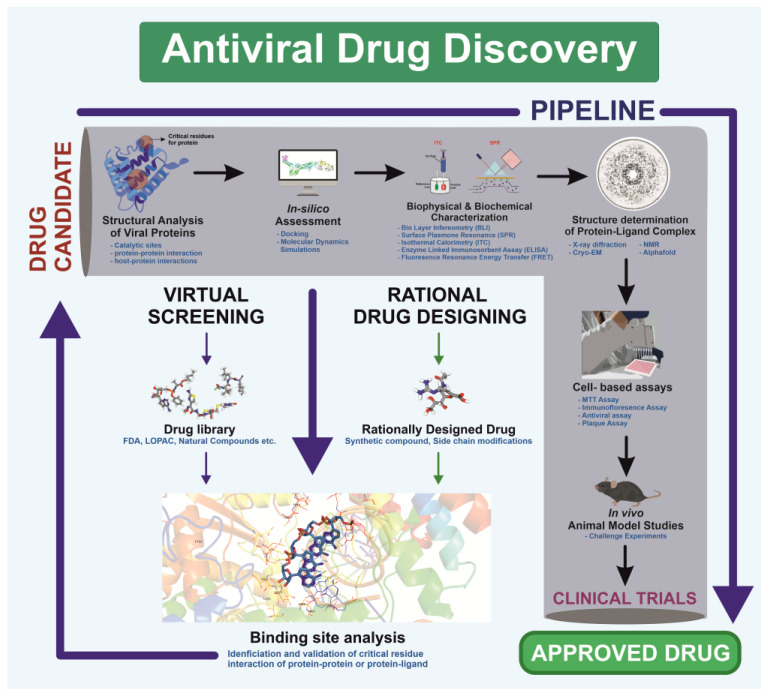
Schematic illustration of the antiviral drug discovery pipeline targeting a viral protein, integrating screening and rational design approaches. The workflow encompasses virtual screening, hit identification, in vitro validation, co-crystallization, cell-based assays, and in vivo efficacy evaluation, ensuring a systematic progression from compound identification to drug development.

Visualization forms the foundation of rational drug design, enabling researchers to analyze viral structures [294]. This structural understanding facilitates precise drug design based on viral protein architecture, as demonstrated during the COVID-19 pandemic [295]. Unlike traditional methods, which relied on screening drugs for antiviral activity without prior molecular insight, rational drug design employs high-resolution structural techniques—X-ray crystallography, NMR spectroscopy, and cryo-EM—to study viral proteins and processes essential for replication [296]. The process begins by identifying a target viral protein critical to replication or host entry. Structural analyses then reveal active sites and binding pockets where small molecules can inhibit protein function [297]. Using this data, scientists design molecules tailored to fit these pockets, thereby blocking protein activity. This targeted strategy improves efficacy and minimizes side effects by avoiding interactions with non-target proteins (Figure 4).

Rational drug design combines structural and biochemical insights to create highly specific therapies, transforming antiviral treatment approaches. For example, the small molecule Nirmatrelvir targets SARS-CoV-2’s main protease, showing high antiviral efficacy [298]. Similarly, analogs like spirolactam, derived from zamnair, improve antiviral potency, while peptides such as VIR250 selectively inhibit the papain-like protease of SARS-CoV-2 [297]. Nanoparticles, like the ICO-RBD nanovaccine, mimic virus-like structures to boost immunogenicity [299]. Future developments in rational drug design promise faster and more precise solutions for emerging pathogens, leveraging computational tools and structural biology advancements.

Rational drug design has transformed structural biology by providing a direct pathway from understanding a virus’s atomic structure to developing effective antiviral therapies [300]. This approach has led to the creation of antiviral drugs for diseases such as HIV/AIDS and influenza, where structural studies of viral enzymes and surface proteins play a key role in designing inhibitors. Additionally, this method is vital for quickly developing treatments against emerging viral threats, as demonstrated by the rapid creation of SARS-CoV-2 spike protein inhibitors during the COVID-19 pandemic. AI has further accelerated this process by reducing screening times for antivirals, improving predictive models for binding affinity, and enabling data mapping to track trends in viral mutations and forecast future changes [301,302]. AI technologies have enhanced drug discovery by optimizing, generating, and identifying molecules with drug-like properties [303]. The need for such advancements in rational drug design is clear, as they support the rapid development of therapeutics to combat future pandemics. Ultimately, rational drug design not only deepens our understanding of drug mechanisms but also advances public health by enabling the swift creation of targeted treatments for both existing and emerging viral diseases [304] (Figure 4).

### De Novo Designing—A Targeted Approach with Improved Features

*De novo* protein design has emerged as a promising strategy for creating molecules from scratch, contributing significantly to combating viral infections [305]. Computational pipelines can be developed and deployed for the streamlined development of antibody-based therapeutic interventions against emerging pathogens [306]. Applications include an in silico affinity maturation pipeline developed and employed to successfully bioengineer nanobodies with enhanced affinity [307]. With the integration of generative AI, it is now possible to engineer proteins targeting specific protein structures. This advancement also enables personalized therapeutic research tailored to individual patients [308,309]. Large language models, such as PALM-H3, further enhance these capabilities by generating antibodies using pre-trained models, allowing for the de novo synthesis of antibodies [310]. Beyond drug design, vaccines targeting viral proteins can also be developed to present surface glycoproteins and elicit immunization responses. Modeling tools such as SabPred assist further in modeling antibodies and in silico validation of structures [311]. For instance, HIV vaccines displaying envelope glycoproteins have demonstrated strong neutralizing titers, emphasizing the effectiveness of nanoparticle-based strategies [312,313]. Moreover, generative AI can be utilized to produce antibodies specific to target antigens, enhancing therapeutic applications [314].

Breakthroughs such as AlphaFold have revolutionized protein modeling, enabling the accurate prediction of protein structures [315]. These tools facilitate the identification of protein functions and their interactions with other molecules [93]. Further advancements, like RFdiffusion, simplify the design of protein binders that target specific antigen sites, eliciting neutralizing responses against viruses [316]. Various approaches taken for designing these antivirals are mentioned in Table 3. A rational approach has not only been evident in designing antivirals but also antibodies and vaccines designing too.

## 7. Identifying the Threat of Future—A Structural Approach

Viruses pose a significant global health threat due to their potential to cause epidemics and pandemics. The WHO R&D Blueprint for Epidemics prioritizes identifying high-risk viruses for early detection, targeted research, efficient resource allocation, and global collaboration (WHO Pathogen Prioritization Report, 2024) [329] (Table 4). “Pathogen X”, an unknown future threat, underscores the need for preparedness. Identifying new pathogens involves robust surveillance, genomic sequencing, and epidemiological studies. Targeting Pathogen X requires focusing on pathogen families, studying prototype pathogens, fostering international collaboration, and investing in R&D [330]. Prioritizing high-risk viruses and preparing for Pathogen X enhances global response capabilities, ultimately saving lives and protecting public health (WHO Pathogen Prioritization Report, 2024) [329]. Knowing the threat, their structures, and a rational approach to designing therapeutics, there is hope of fighting the upcoming pandemics with advanced knowledge of structural virology.

## 8. Conclusions and Future Direction

Structural virology has played an instrumental role in shaping our understanding of viral mechanisms and enabling the development of targeted therapeutics. Key techniques like X-ray crystallography, cryo-EM, and NMR spectroscopy have yielded high-resolution images of viral structures, revealing key molecular targets on enzymes, receptors, and structural proteins. With the ever-increasing data and research on virus-encoded proteins, structural biology has proven to be an indispensable tool for rational design and optimization of antiviral drugs. Additionally, computational methods have become integral to the early stages of drug discovery and development. The review delves into existing and new technological advancements to further the depth of structural understanding of viral proteins, interactions, and the strategies for antiviral therapeutic development.

Although notable strides have been made in the field, the structural data of many of the key proteins are yet to be elucidated. Therefore, it is imperative to develop strategies for obtaining structural data and establishing a link between structure and function. COVID-19 demonstrated the significance of pandemic preparedness for the world. Based on historical precedents of pandemics and epidemics that have plagued the world, debilitated global healthcare systems, and caused substantial mortality and lasting health impact. COVID-19 is unlikely to be the final pandemic. Therefore, continuous efforts from the scientific community to develop antiviral therapeutics against emerging pathogens and pathogens with pandemic potential is the need of the hour. Further investigations are crucial for identifying druggable host factors for the development of broad-spectrum therapeutics.

Due to rapid rate of mutation, many viruses are emerging and reemerging pathogens. Mutation prediction, leveraging sequence and structural data along with surveillance data are now critical in forecasting evolution, aiding to guide the design of adaptive therapeutic strategies that can keep up with the rapid rate of viral evolution. As witnessed during the SARS-CoV-2 pandemic, available and ongoing research to elucidate the protein structures expedited vaccine development, whereas the rapid identification of variants underscored the importance of anticipating viral evolution in real-time. In the future, real-time structural surveillance, coupled with AI-powered tools, will be pivotal in swiftly identifying mutations and new pathogens. It will be essential to assess the impact of viral mutations on transmissibility and immune escape. Considering the rise of emerging and reemerging viruses, it is crucial to identify the viruses that may pose a threat to our health in the future and to develop strategies to address them before they escalate into a severe pandemic. Consequently, numerous global organizations, such as the WHO, have recognized several of these viruses and have initiated efforts to address them. Having a strategic plan in place to address the threat will provide greater safety for dealing with future viral challenges.

## Figures and Tables

**Table 1:** List of FDA-approved therapeutics against viral structural proteins.

Enveloped Glycoprotein						
Protein Name	Virus (Family)	Name of Therapeutic	Type	Brand Name	Experimental Method	PDB ID
N1 neuraminidase	Influenza A Virus (*Orthomyxoviridae*)	Zanamivir	Antiviral	Relenza	X-ray diffraction	3CKZ,
N8 neuraminidase	Influenza A Virus (*Orthomyxoviridae*)	Zanamivir	Antiviral	Relenza	X-ray diffraction	2HTQ
N1 Neuraminidase	Influenza A Virus (*Orthomyxoviridae*)	Oseltamivir	Antiviral	Tamiflu	X-ray diffraction	3CL0, 2HU4
Hemagglutinin (HA)	Influenza A Virus (*Orthomyxoviridae*)	Umifenovir	Antiviral	Arbidol	X-ray diffraction	5T6N, 5T6S
Neuraminidase (NA)	Influenza A Virus (*Orthomyxoviridae*)	Laninamivir octanoate	Antiviral	Inavira	X-ray diffraction	3TI4
N8 neuraminidase	Influenza A Virus (*Orthomyxoviridae*)	Peramivir	Antiviral	Rapivab	X-ray diffraction	2HTU
Glycoprotein 120	HIV-1 (*Retroviridae*)	412d	Antibody (Human)	N/A	X-ray diffraction	2QAD
glycoprotein (GP) trimer	Ebola virus (*Filoviridae*)	Atoltivimab, maftivimab and odesivimab	Antibody (Human)	Inmazeb (REGN-EB3)	Cryo-EM	7TN9
glycoprotein (GP) trimer	Ebola virus (*Filoviridae*)	mAb100 and mAb114	Antibody (Human)	Ansuvimab	X-ray diffraction	5FHC
Spike protein (S)	SARS-CoV-2 (*Coronaviridae*)	LY-CoV555	Antibody (Human)	Bamlanivimab	X-ray diffraction	7KMG
Spike RBD	SARS-CoV-2 (*Coronaviridae*)	Ly-Cov1404	Antibody (Human)	Bebtelovimab	X-ray diffraction	7MMO
Spike RBD	SARS-CoV-2 (*Coronaviridae*)	azd8895 and azd1061	Antibody (Human)	Cilgavimab	X-ray diffraction	7L7E
Spike RBD	SARS-CoV-2 (*Coronaviridae*)	CA1 and CB6	Antibody (Human)	Etesevimab	X-ray diffraction	7C01
Spike RBD	SARS-CoV-2 (*Coronaviridae*)	REGN10933 and REGN10987	Antibody (Human)	Masavibart	Cryo-EM	6XDG
Spike RBD	SARS-CoV-2 (*Coronaviridae*)	Ct-P59	Antibody (Human)	Regdanvimab	X-ray diffraction	7CM4
Spike RBD	SARS-CoV-2 (*Coronaviridae*)	GAR05 and GAR12	Antibody (Human)	Sotrovimab	X-ray diffraction	7T72
Spike RBD	SARS-CoV-2 (*Coronaviridae*)	DMAbs 2130 and DMAbs 2196	Antibody (Human)	Tixagevimab	Cryo-EM	8D8Q
Spike protein (S)	SARS-CoV-2 (*Coronaviridae*)	REGN10933 and REGN10987	Antibody (Human)	REGEN-COV	X-ray diffraction	7M42
Fusion Glycoprotein (F)	Respiratory syncytial virus (RSV)	MEDI8897	Antibody (Human)	Palivizumab	X-ray diffraction	5UDC
gp41 subunit (Envelope glycoprotein)	HIV-1	T20	Peptide	Enfuvirtide	X-ray diffraction	5ZCX
Capsid Protein						
Protein Name	Virus (Family)	Name of Therapeutic	Type	Brand Name	Experimental Method	PDB ID
HBV Capsid	hepatitis B virus (HBV) (*Picornaviridae*)	Lenacapavir (DBT1)	Antiviral	Sunlenca	Cryo-EM	6WFS
HBV capsid assembly	hepatitis B virus (HBV) (*Picornaviridae*)	AT-130	Antiviral	-	X-ray diffraction	4G93
HBV capsid assembly	hepatitis B virus (HBV) (*Picornaviridae*)	HAP18	Antiviral	-	X-ray diffraction	5D7Y
Membrane Proteins						
Protein Name	Virus (Family)	Name of Therapeutic	Type	Brand Name	Experimental Method	PDB ID
Membrane protein (M2)	Influenza A Virus (*Orthomyxoviridae*)	Amantadineb	Antiviral	-	X-ray diffraction	3C9J
Membrane protein (M2)	Influenza A Virus (*Orthomyxoviridae*)	Rimantadine	Antiviral	Flumadine	X-ray diffraction	6BKL, 6US9

**Table 2:** List of FDA-approved drugs targeting viral replication enzymes.

Viral Replication Enzymes						
Target Protein		Virus (Family)	Drug	Brand Name	Experimental Method	PDB ID
Protease	HIV protease	HIV-1 and HIV-2 (*Retroviridae*)	Saquinavir (SQV)	Invirase ^a^	X-ray diffraction	1HXB
			Ritonavir (RTV)	Norvir	X-ray diffraction	1HXW
			Indinavir (IDV)	Crixivan ^a^	X-ray diffraction	1HSG
			Nelfinavir (NFV)	Viracept	X-ray diffraction	1OHR
			Lopinavir (LPV)	Kaletra, (combination with ritonavir)	X-ray diffraction	1MUI
			Atazanavir (ATV)	Reyataz	X-ray diffraction	2AQU
			Darunavir (DRV)	Prezista	X-ray diffraction	1T3R
		HIV-1 (*Retroviridae*)	Amprenavir (APV)	Agenerase ^a^	X-ray diffraction	1HPV
					Neutron diffraction	4JEC
			Tipranavir (TPV)	Aptivus	X-ray diffraction	1D4S
	HCV NS3/4A protease	HCV genotype 1 (*Flaviviridae*)	Telaprevir (TVR)	Incivek ^a^	X-ray diffraction	3SV6
			Boceprevir (BOC)	Victrelis ^a^	X-ray diffraction	3LOX
			Simeprevir (SMV)	Olysio ^a^	X-ray diffraction	3KEE
			Vaniprevir (VPV)	Vanihep, in combination with ribavirin + PegIFNα-2b	X-ray diffraction	3SU3
		HCV genotype 1 and 4 (*Flaviviridae*)	Asunaprevir (ASV)	Sunvepra ^b^	X-ray diffraction	4WF8
			Grazoprevir (GZR)	Zepatier	X-ray diffraction	3SUD
		HCV genotype 1 to 4 (*Flaviviridae*)	Voxilaprevir (VOX)	Vosevi	X-ray diffraction	6NZT
			Glecaprevir (GLE)	Mavyret	X-ray diffraction	6P6L
	Main proteases (M pro or 3-chymotrypsin-like proteases (3CL pro)	SARS-CoV-2 (*Coronaviridae*)	Nirmatrelvir (NMV)	Paxlovid (combination with ritonavir)	X-ray diffraction	7SI9
			Ensitrelvir (ENS)	Xocova ^b^	X-ray diffraction	7VU6
Polymerase	DNA-dependent DNA polymerase (DdDp)	HSV (*Herpesviridae*)	Foscarnet (PFA)	Foscavir	Cryo-EM	8EXX
			Acyclovir (ACV)	Zovirax	Cryo-EM	8V1T
	Reverse Transcriptase (RT)	HIV-1 and HIV-2 (*Retroviridae*)	Stavudine (d4T)	Zerit ^a^	X-ray diffraction	6AMO
			Lamivudine (3TC)	Epivir, Combivir (combination with Zidovudine), Trizivir (combination with Zidovudine and abacavir)	X-ray diffraction	6KDJ
			Tenofovir disoproxil fumarate (TDF)	Viread	X-ray diffraction	1T05
			Doravirine (DOR)	Pifeltro	X-ray diffraction	4NCG
					Cryo-EM	7Z2G
		HIV-1 (*Retroviridae*)	Nevirapine (NVP)	Nevirapine (generic)	X-ray diffraction	1FKP
					Cryo-EM	7KJX
			Delavirdine (DLV)	Rescriptor ^a^	X-ray diffraction	1KLM
			Efavirenz (EFV)	Efavirenz (generic)	X-ray diffraction	1FK9
					Cryo-EM	7KJW
			Etravirine (ETR)	Intelence	X-ray diffraction	1SV5
			Rilpivirine (RPV)	Edurant	X-ray diffraction	2ZD1
					Cryo-EM	7Z2D
			Dapivirine (DPV)	DapiRing ^b^	X-ray diffraction	1S6Q
	RNA-dependent RNA polymerase (RdRp)	Influenza A and B viruses (*Orthomyxoviridae*)	Baloxavir (BXA)	Xofluza	X-ray diffraction	6FS6
		SARS-CoV-2 (*Coronaviridae*)	Remdesivir (RDV)	Veklury	Cryo-EM	7BV2
			Molnupiravir (MOV)	Lagevrio ^b,c^	Cryo-EM	7OZU
			Favipiravir (FVP)	Avigan ^b^	Cryo-EM	7CTT
Integrase (IN)	Retroviral IN	HIV-1 and HIV-2 (*Retroviridae*)	Dolutegravir (DTG)	Tivicay, Triumeq (combination), Dutrebis (combination)	X-ray diffraction	3S3M
					Cryo-EM	8FN7
			Bictegravir (BIC)	Biktarvy (combination)	Cryo-EM	6PUW
Thymidine kinase (TK)	HSV-1 TK	HSV-1 (*Herpesviridae*)	Idoxuridine (IDU) -5-substituted 2′-deoxyuridine analog, substrate of TK	Dendrid	X-ray diffraction	1KI7
			Brivudine (BVDU) 5-substituted 2′-deoxyuridine analog, substrate of TK	Zostex ^b^	X-ray diffraction	1KI8
		HSV (*Herpesviridae*)	Penciclovir (PCV)	Denavir	X-ray diffraction	1KI3
			Acyclovir (ACV)	Zovirax	X-ray diffraction	2KI5
	VZV TK	VZV (*Herpesviridae*)	Brivudine (BVDU) 5-substituted 2′-deoxyuridine analog, substrate of TK	Zostex ^b^	X-ray diffraction	1OSN

^a^ Discontinued. ^b^ Approved in some countries, not FDA-approved. ^c^ Emergency use authorization (EUA) by FDA.

**Table 3:** List of therapeutics and the rational approaches used in their designing.

Name of Therapeutic	Target Virus (Family)	Rational Designing Approach and Result	Type	PDB	Ref.
Nirmatrelvir	SARS-CoV-2	A protease inhibitor for SARS-CoV-2 with reported protein–ligand complex, demonstrating its binding to the viral main protease (Mpro), which is crucial for viral replication.	Ligand	7TLL	[298]
Spirolactam	Influenza A	Carbocyclic analog of zanamivir in which the hydrophilic glycerol side chain is replaced by the hydrophobic 3-pentyloxy group of oseltamivir. This hybrid inhibitor showed excellent inhibitory properties in the neuraminidase inhibition assay	Ligand	4MJV	[317]
VIR 250 and VIR 251	SARS-CoV-2	Designed and biochemically characterized potent inhibitors (VIR250 and VIR251) harboring high selectivity for SARS-CoV-2 PLpro and the related SARS-CoV-1 PLpro versus other proteases.	Peptide	6WUU, 6WX4	[297]
Adintrevimab	SARS-CoV-2	The crystallizable fragment (Fc) region of adintrevimab undergoes affinity maturation, leading to two amino acid modifications (S52A and W100B) that enhance the half-life while preserving the normal effector functions of IgG1.	Antibody	7U2E	[318]
RBD-I53-50 nanoparticles	SARS-CoV-2	The nanoparticle vaccines present 60 SARS-CoV-2 spike receptor-binding domains (RBDs) were arranged in a highly immunogenic configuration, yielding neutralizing antibody titers tenfold greater than those elicited by the prefusion-stabilized spike, even when administered at a fivefold lower dosage.	Nanoparticle Vaccine	-	[319]
ICO-RBD nanovaccine	SARS-CoV-2	The immunogenicity of nanovaccines aimed at viral components, including the SARS-CoV-2 receptor-binding domain (RBD), is improved by multivalent antigen presentation on nanoparticles. Using icosahedral DNA origami (ICO) as a display particle, we achieve virus-like morphology and diameter for RBD nanovaccines.	Nanoparticle Vaccine	-	[299]
GPC-I53-50NP	Lassa virus (LASV)	The study employs two-component protein nanoparticles to stabilize the Lassa virus glycoprotein complex (GPC) in its trimeric form. These nanoparticles elicited robust antibody responses in rabbits and provided protection to guinea pigs against lethal LASV challenge experiments.	Nanoparticle Vaccine	7SGE	[320]
D25	Respiratory syncytial virus (RSV)	To elicit robust and specific neutralizing antibodies (nAbs), immunogens were designed with a focus on the epitopes derived from the prefusion structure of the respiratory syncytial virus (RSV) fusion protein.	Antibody	-	[321]
DS-Cav1-I53-50	Respiratory syncytial virus (RSV)	The approach involves presenting a prefusion-stabilized variant of the F glycoprotein trimer (DS-Cav1) in a repetitive array on the nanoparticle exterior. This two-component nanoparticle scaffold enables the production of highly ordered monodisperse immunogens that display DS-Cav1 at controllable density.	Nanoparticle Vaccine	-	[313]
Fab 14N4	Respiratory syncytial virus (RSV)	Palivizumab is a humanized monoclonal antibody derived from a murine mAb, designed to target antigenic site II of the RSV fusion (F) protein, a critical target in vaccine development.	Antibody	5J3D	[322]
Casirivimab and Imdevimab	SARS-CoV-2	This cocktail, targeting the SARS-CoV-2 spike protein‘s receptor-binding domain, received Emergency Use Authorization (EUA) from the FDA in November 2020 for COVID-19 treatment, effectively preventing viral mutation escape.	Antibody	6XDG	[323]
Bamlanivimab and Etesevimab	SARS-CoV-2	A combination targeting the SARS-CoV-2 spike protein was granted EUA in February 2021. These rapidly developed monoclonal antibodies have proven effective in reducing viral load and improving patient outcomes. The Fc region was modified to enhance stability.	Antibody	7KMG, 7C01	[324]
Tixagevimab and Cilgavimab	SARS-CoV-2	This combination, targeting SARS-CoV-2, received EUA for COVID-19 prevention and treatment and includes engineered Fc domains to reduce adverse effects.	Antibody	8D8Q, 7L7E	[325]
Sotrovimab	SARS-CoV-2	B-cells from a SARS-CoV-infected individual, with an Fc domain modification to extend half-life, received FDA EUA in 2021 for treating mild-to-moderate COVID-19.	Antibody	-	[326]
Spike protein Epitope-scaffolds ^a^	SARS-CoV-2	This study introduces a novel vaccine design strategy targeting conserved regions of the SARS-CoV-2 spike protein, bypassing the mutagenic receptor-binding domain. Using epitope grafting, stable immunogens mimicking these regions‘ surface features were engineered. Immunogenicity assessments in murine models showed promising results, and the designed epitope-scaffolds demonstrated potential diagnostic utility.	Nanoparticle Vaccine	-	[327]
VHH60	SARS-CoV-2	Derived from the FDA-approved nanobody caplacizumab, this neutralizing nanobody binds the receptor-binding domain of the SARS-CoV-2 spike protein with high affinity (2.56 nM). It has shown significant efficacy in inhibiting virus infection in vitro and in vivo, making it a strong candidate for further clinical investigation against COVID-19.	Nanobody	-	[328]

^a^ Not clinically approved.

**Table 4:** Virus families and pathogens identified as high-priority Public Health Emergencies of International Concern (PHEICs) for 2024. The corresponding PDB IDs of structural and non-structural proteins for these “high-risk” viruses are provided, highlighting potential antiviral therapeutic targets. (Source: WHO Pathogen Prioritization Report, 2024).

Family	Priority Pathogens	Virus	Viral Protein	PDB ID
Arenaviridae	*Mammarenavirus lassaense*	Lassa virus	Glycoprotein Complex (GPC)	8TYE
			Nucleoprotein	3MWP
			matrix protein Z	5I72
			spike complex	7PVD
			L protein	7OJJ
	*Mammarenavirus juninense*	Junin virus	GP1 glycoprotein	5NUZ
			Nucleoprotein	4K7E
			L protein	7EJU
			Z protein	7EJU
	*Mammarenavirus lujoense*	Lujo Virus	spike complex	8P4T
			GP1 domain	6GH8
	*Mamastrovirus virginiaense*	Astrovirus	capsid spike	7RK2
*Coronaviridae*	Subgenus Merbecovirus	MERS	Spike Protein	8SAK
			Fusion core	4MOD
			Papain-like Potease	4P16
			3CL Protease	4WMD
			nsP1	8T4S
			nsp5 protease	4YLU
			nsP10	5YN5
			nsP13	5WWP
			nsP16	5YN5
			Nucleocapsid N-terminal Domain (NTD)	4UD1
			Nucleocapsid C-terminal Dmain	7DYD
	Subgenus Sarbecovirus	SARS-CoV-2	Spike Protein	6M0J
			Envelope Protein	8U1T
			Membrane protein	8CTK
			Nucleocapsid N-terminal Domain (NTD)	7ACT
			Nucleocapsid C-terminal Dmain	7O05
			nsP1	7K3N
			nsP2	7MSX
			nsP3 (papain-like Protease)	6W9C
			nsP5 (Main protease)	6y2e
			nsP7	7JLT
			nsP8	7JLT
			nsP9	6WXD
			nsP10	5YN5
			nsP12	6NUR
			nsp13	6ZSL
			nsp14	5C8S
			nsp15	6VWW
			nsp16	5YN5
*Flaviviridae*	*Orthoflavivirus zikaense*	Zika Virus	Envelope Protein	5JHM,
			Membrane Protein	7KCR
			NS5 Methyltransferase	5GP1
	*Orthoflavivirus denguei*	Dengue Virus	Envelope Protein	1K4R, 7BUD
			Membrane Protein	7BUD
			NS3 protein	5YVW
			NS2B/NS3 Protease	2FOM
			NS5	8T12
	*Orthoflavivirus encephalitidis*	Japanese encephalitis virus	Envelope Protein	3P54
			Capsid	5OW2
			Membrane Protein	5WSN
			Nsp1 CTD	5O36
			NS5	4K6M
	*Orthohantavirus sinnombreense*	Sin Nombre virus	Envelope Protein (Gc)	7FGF
			Envelope Protein (Gn)	8AHN
			Nucleocapsid	2IC9
*Orthomyxoviridae*	*Alphainfluenzavirus Influenzae H1*	Influenza A Virus	Hemagglutinin H1	6CHX
			Hemagglutinin H5	5Z88
			Neuraminidase	4HZY
			RNA Polymerase	6QPG
*Paramyxoviridae*	*Henipavirus nipahense*	Nipah Virus	Fusion Core	1WP7
			Glycoprotein	2VSM
			Matrix Protein	7SKT
			Nucleoprotein	4CO6
			Phosphoprotein	7PNO
			Polymerase	9IR3
*Poxviridae*	*Orthopoxvirus variola*	Variola virus	Phosphoprotein	6EB9
			L1 protein	1YPY
			Topoisomerase	3IGC
	*Orthopoxvirus vaccinia*	Vaccinia Virus	DNA-dependent RNA polymerase complex	6RFL
			virulence factor F1L	5AJJ
	*Orthopoxvirus monkeypox*	Monkeypox Virus	H3 envelope protein	5EJ0
			methyltransferase VP39	8B07
*Togaviridae*	*Alphavirus chikungunya*	Chikungunya virus	E1 and E2 Envelope Glycoproteins	2XFB
			Capsid Protein	5H23
			nsP2 Protease	4ZTB
			nsP3	4TU0
			nsP4	7VB4
	*Alphavirus venezuelan*	Venezuelan Equine Encephalitis virus (VEEV)	Envelope Glycoprotein	7SFV
			nsP2 Protease	5EZQ

## Abbreviations

The following abbreviations are used in this manuscript:

3CLpro	3-chymotrypsin-like proteases
3D	Three-dimensional
3-P	3′-processing
3TC	Lamivudine
5-CITEP	1-(5-chloroindol-3-yl)-3-hydroxy-3-(2H-tetrazol-5-yl)propenone
ABT	ABT199/venetoclax
ACE2	Angiotensin-converting enzyme 2
ACV	Acyclovir
ACV	Acyclovir
ADE	Antibody-dependent enhancement
AI	Artificial Intelligence
ALLINI	Allosteric IN inhibitors
APV	Amprenavir
ART	Antiretroviral therapy
ASV	Asunaprevir
ATV	Atazanavir
BH	Berbamine hydrochloride
BIC	Bictegravir
BOC	Boceprevir
BVDU	Brivudine
BXA	Baloxavir
CAPE	Caffeic acid phenethyl ester
CAR	Coxsackievirus and Adenovirus Receptor
cART	Combination antiretroviral therapy
CCR5	C-C chemokine receptor type 5
CD4	Cluster of differentiation 4
CEN	Cap-dependent endonuclease
CHIKV	Chikungunya Virus
CLR	C-type lectin receptor
CMV	Cucumber Mosaic Virus
COVID-19	Coronavirus disease 2019
CRISPR	Clustered Regularly Interspaced Short Palindromic Repeats
cryo-EM	Cryo-electron microscopy
Cryo-ET	Cryo-electron tomography
CsA	Cyclosporin A
CVB4	Coxsackie B4 virus
CXCR4	C-X-C chemokine receptor type 4
Cyp	Cyclophilin
d4T	Stavudine
DC-SIGN	Dendritic Cell-Specific Intercellular adhesion molecule-3-Grabbing Non-integrin
DdDp	DNA-Dependent DNA Polymerase
DdRp	DNA-Dependent RNA Polymerase
DENV	Dengue Virus
DKA	Diketoacid
DLV	Delavirdine
DNA	Deoxyribonucleic Acid
DOR	Doravirine
DPV	Dapivirine
DRV	Darunavir
dT	Deoxythymidine
DTG	Dolutegravir
EFV	Efavirenz
ENS	Ensitrelvir
ETR	Etravirine
EUA	Emergency use authorization
FDA	Food and Drug Administration
FVP	Favipiravir
G3BP1	Ras GTPase-activating protein SH3-domain-binding protein 1
GLE	Glecaprevir
GTase	m7GTP transferase
GV	Giant viruses
GZR	Grazoprevir
HA	hemagglutinin
HBV	Hepatitis B Virus
HC	Herbacetin
HCoV	Human Coronavirus-
HCV	Hepatitis C Virus
HHV	Human Herpesvirus
HIV	Human Immunodeficiency Virus
HPV	Human Papillomavirus
HS	heparan sulfate
HTLV	Human T-Cell Leukemia Virus
IBD	Integrase binding domain
ICTV	International Committee on Taxonomy of Viruses
IDP	Intrinsically disordered proteins
IDR	Intrinsically disordered regions
IDU	Idoxuridine
IDV	Indinavir
IFN	Interferon
IFN	Interferon
IL-10	Interleukin 10
IN	Integrase
INSTI	Integrase strand transfer inhibitors
INT	Intasome
IQM	Imiquimod
IR	Infrared
ISG	Interferon-Stimulated Genes
KSHV	Kaposi‘s Sarcoma-Associated Herpesvirus
LEDGF/p75	Lens Epithelium-Derived Growth Factor/p75
LPV	Lopinavir
MCV or MCPyV	Merkel Cell Polyomavirus
MD	Molecular Dynamics
MERS-CoV	Middle East respiratory syndrome coronavirus
MINI	Multimerization-selective integrase inhibitor
MOV	Molnupiravir
Mpro	Main protease
MPXV	Monkeypox virus
MTase	Methyltransferase
MXRA8	Matrix remodeling-associated protein 8
NA	Neuraminidase
NFV	Nelfinavir
NiRAN	RdRp-associated nucleotidyltransferase
NMR	Nuclear Magnetic Resonance
NMV	Nirmatrelvir
NNRTI	Non-nucleoside reverse transcriptase inhibitors
NRTI	Nucleoside reverse transcriptase inhibitors
NTD	N-terminal domain
NTD	N-terminal domain
NTP	Nucleoside triphosphate
NVP	Nevirapine
PCV	Penciclovir
PDX	Podofilox
PFA	Foscarnet
PI	Peptide inhibitors
PIC	Pre-integration complex
PLpro	Papain-like protease
PPI	Protein–protein interactions
PRF	Programmed ribosomal frameshifting
PT	Ponatinib
PVY	Potato Virus Y
RABV	Rabies Virus
RBD	Receptor-binding domain
RdRp	RNA-Dependent RNA Polymerase
RDV	Remdesivir
RNA	Ribonucleic Acid
RNase	Ribonuclease
RNP	Ribonucleoprotein
RPV	Rilpivirine
RSV	Respiratory syncytial virus
RT	Reverse Transcriptase
RTV	Ritonavir
RV	Rotavirus
SAH	S-adenosyl homocysteine
SAM	S-adenosyl-l-methionine
SARS-CoV	Severe Acute Respiratory Syndrome Coronavirus
SARS-CoV-2	Severe Acute Respiratory Syndrome Coronavirus 2
SAXS	Small Angle X-ray Scattering
SBDD	Structure-based drug design
SERM	Selective estrogen receptor modulators
SG	Stress granule
SINE	Sinecatechins
SINV	Sindbis Virus
SMV	Simeprevir
SQV	Saquinavir
ST	Strand transfer
TBSV	Tomato bushy stunt virus
TDF	Tenofovir disoproxil fumarate
TEM	Transmission electron microscopy
TK	Thymidine kinase
TMV	Tobacco Mosaic Virus
TPV	Tipranavir
TVR	Telaprevir
VEEV	Venezuelan Equine Encephalitis virus
VLP	Virus-like particle
VOX	Voxilaprevir
VPV	Vaniprevir
VZV	Varicella-zoster virus
WHO	World Health Organization
XFEL	X-ray Free Electron Laser
XRD	X-ray diffraction

## References

[1] Payne S. (2017). Introduction to Animal Viruses. Viruses.

[2] Current ICTV Taxonomy Release|ICTV. https://ictv.global/taxonomy.

[3] Wirth J., Young M. (2020). The Intriguing World of Archaeal Viruses. PLoS Pathog..

[4] Krupovic M., Cvirkaite-Krupovic V., Iranzo J., Prangishvili D., Koonin E.V. (2018). Viruses of Archaea: Structural, Functional, Environmental and Evolutionary Genomics. Virus Res..

[5] Mahler M., Costa A.R., van Beljouw S.P.B., Fineran P.C., Brouns S.J.J. (2023). Approaches for Bacteriophage Genome Engineering. Trends Biotechnol..

[6] Gordillo Altamirano F.L., Barr J.J. (2019). Phage Therapy in the Postantibiotic Era. Clin. Microbiol. Rev..

[7] Queiroz V.F., Tatara J.M., Botelho B.B., Rodrigues R.A.L., Almeida G.M.d.F., Abrahao J.S. (2024). The Consequences of Viral Infection on Protists. Commun. Biol..

[8] Kalafati E., Papanikolaou E., Marinos E., Anagnou N.P., Pappa K.I. (2022). Mimiviruses: Giant viruses with novel and intriguing features. Mol. Med. Rep..

[9] Coy S.R., Gann E.R., Pound H.L., Short S.M., Wilhelm S.W. (2018). Viruses of Eukaryotic Algae: Diversity, Methods for Detection, and Future Directions. Viruses.

[10] Hough B., Steenkamp E., Wingfield B., Read D. (2023). Fungal Viruses Unveiled: A Comprehensive Review of Mycoviruses. Viruses.

[11] Jones R.A.C., Janssen D. (2021). Global Plant Virus Disease Pandemics and Epidemics. Plants.

[12] Tatineni S., Hein G.L. (2023). Plant Viruses of Agricultural Importance: Current and Future Perspectives of Virus Disease Management Strategies. Phytopathology.

[13] Bertola M., Mutinelli F. (2021). A Systematic Review on Viruses in Mass-reared Edible Insect Species. Viruses.

[14] Garrison A.R., Alkhovsky S.V., Avšič-Županc T., Bente D.A., Bergeron É., Burt F., Paola N.D., Ergünay K., Hewson R., Kuhn J.H. (2020). ICTV Virus Taxonomy Profile: Nairoviridae. J. Gen. Virol..

[15] Simmonds P., Becher P., Bukh J., Gould E.A., Meyers G., Monath T., Muerhoff S., Pletnev A., Rico-Hesse R., Smith D.B. (2017). ICTV Virus Taxonomy Profile: Flaviviridae. J. Gen. Virol..

[16] Bolling B.G., Weaver S.C., Tesh R.B., Vasilakis N. (2015). Insect-Specific Virus Discovery: Significance for the Arbovirus Community. Viruses.

[17] Javanian M., Barary M., Ghebrehewet S., Koppolu V., Vasigala V.K.R., Ebrahimpour S. (2021). A Brief Review of Influenza Virus Infection. J. Med. Virol..

[18] Hutchinson E.C. (2018). Influenza Virus. Trends Microbiol..

[19] Brunker K., Mollentze N. (2018). Rabies Virus. Trends Microbiol..

[20] Li G., Hilgenfeld R., Whitley R., De Clercq E. (2023). Therapeutic Strategies for COVID-19: Progress and Lessons Learned. Nat. Rev. Drug Discov..

[21] Ciotti M., Ciccozzi M., Terrinoni A., Jiang W.C., Wang C.B., Bernardini S. (2020). The COVID-19 Pandemic. Crit. Rev. Clin. Lab. Sci..

[22] Crimi S., Fiorillo L., Bianchi A., D’amico C., Amoroso G., Gorassini F., Mastroieni R., Marino S., Scoglio C., Catalano F. (2019). Herpes Virus, Oral Clinical Signs and QoL: Systematic Review of Recent Data. Viruses.

[23] Rechenchoski D.Z., Faccin-Galhardi L.C., Linhares R.E.C., Nozawa C. (2017). Herpesvirus: An Underestimated Virus. Folia Microbiol..

[24] Connolly S.A., Jardetzky T.S., Longnecker R. (2020). The Structural Basis of Herpesvirus Entry. Nat. Rev. Microbiol..

[25] Agut H., Bonnafous P., Gautheret-Dejean A. (2015). Laboratory and Clinical Aspects of Human Herpesvirus 6 Infections. Clin. Microbiol. Rev..

[26] de Sanjosé S., Brotons M., Pavón M.A. (2018). The Natural History of Human Papillomavirus Infection. Best Pract. Res. Clin. Obstet. Gynaecol..

[27] Burd E.M., Dean C.L. (2016). Human Papillomavirus. Diagnostic Microbiology of the Immunocompromised Host.

[28] Schiffman M., Doorbar J., Wentzensen N., De Sanjosé S., Fakhry C., Monk B.J., Stanley M.A., Franceschi S. (2016). Carcinogenic Human Papillomavirus Infection. Nat. Rev. Dis. Primers.

[29] Yoshimura K. (2017). Current Status of HIV/AIDS in the ART Era. J. Infect. Chemother..

[30] Bekker L.G., Beyrer C., Mgodi N., Lewin S.R., Delany-Moretlwe S., Taiwo B., Masters M.C., Lazarus J.V. (2023). HIV Infection. Nat. Rev. Dis. Primers.

[31] Lévêque N., Semler B.L. (2015). A 21st Century Perspective of Poliovirus Replication. PLoS Pathog..

[32] Marzi A., Blanco J.R., Gibellini D., Mbani C.J., Pandoua Nekoua M., Moukassa D., Hober D. (2023). The Fight against Poliovirus Is Not Over. Microorganisms.

[33] Cao J., Li D. (2018). Searching for Human Oncoviruses: Histories, Challenges, and Opportunities. J. Cell Biochem..

[34] Noguera Z.L.P., Charypkhan D., Hartnack S., Torgerson P.R., Rüegg S.R. (2022). The Dual Burden of Animal and Human Zoonoses: A Systematic Review. PLoS Negl. Trop. Dis..

[35] Zeller M.A., Carnevale de Almeida Moraes D., Ciacci Zanella G., Souza C.K., Anderson T.K., Baker A.L., Gauger P.C. (2024). Reverse Zoonosis of the 2022–2023 Human Seasonal H3N2 Detected in Swine. npj Viruses.

[36] Lv J.X., Liu X., Pei Y.Y., Song Z.G., Chen X., Hu S.J., She J.L., Liu Y., Chen Y.M., Zhang Y.Z. (2024). Evolutionary Trajectory of Diverse SARS-CoV-2 Variants at the Beginning of COVID-19 Outbreak. Virus Evol..

[37] Piret J., Boivin G. (2021). Pandemics Throughout History. Front. Microbiol..

[38] Taubenberger J.K., Morens D.M. (2010). Influenza: The Once and Future Pandemic. Public Health Rep..

[39] Eisinger R.W., Fauci A.S. (2018). Ending the HIV/AIDS Pandemic. Emerg. Infect. Dis..

[40] De Cock K.M., Jaffe H.W., Curran J.W. (2021). Reflections on 40 Years of AIDS. Advances in Clinical Immunology, Medical Microbiology, COVID-19, and Big Data.

[41] Chan-Yeung E.M., Xu R., Chan-Yeung M., Chan-yeung M. (2003). SARS: Epidemiology. Respirology.

[42] Raj V.S., Osterhaus A.D.M.E., Fouchier R.A.M., Haagmans B.L. (2014). MERS: Emergence of a Novel Human Coronavirus. Curr. Opin. Virol..

[43] Saha A., Choudhary S., Walia P., Kumar P., Tomar S. (2025). Transformative Approaches in SARS-CoV-2 Management: Vaccines, Therapeutics and Future Direction. Virology.

[44] Listings of WHO’s Response to COVID-19. https://www.who.int/news/item/29-06-2020-covidtimeline.

[45] Duggan A.T., Perdomo M.F., Piombino-Mascali D., Marciniak S., Poinar D., Emery M.V., Buchmann J.P., Duchêne S., Jankauskas R., Humphreys M. (2016). 17th Century Variola Virus Reveals the Recent History of Smallpox. Curr. Biol..

[46] Bandyopadhyay A.S., Garon J., Seib K., Orenstein W.A. (2015). Polio Vaccination: Past, Present and Future. Future Microbiol..

[47] Jacob S.T., Crozier I., Fischer W.A., Hewlett A., Kraft C.S., de La Vega M.A., Soka M.J., Wahl V., Griffiths A., Bollinger L. (2020). Ebola Virus Disease. Nat. Rev. Dis. Primers.

[48] Guo C., Zhou Z., Wen Z., Liu Y., Zeng C., Xiao D., Ou M., Han Y., Huang S., Liu D. (2017). Global Epidemiology of Dengue Outbreaks in 1990–2015: A Systematic Review and Meta-Analysis. Front. Cell Infect. Microbiol..

[49] Douam F., Ploss A. (2018). Yellow Fever Virus: Knowledge Gaps Impeding the Fight Against an Old Foe. Trends Microbiol..

[50] Baud D., Gubler D.J., Schaub B., Lanteri M.C., Musso D. (2017). An Update on Zika Virus Infection. Lancet.

[51] Perry R.T., Halsey N.A. (2004). The Clinical Significance of Measles: A Review. J. Infect. Dis..

[52] Weibel Galluzzo C., Kaiser L., Chappuis F. (2015). Reemergence of Chikungunya Virus. Rev. Med. Suisse.

[53] Turtle L., Solomon T. (2018). Japanese Encephalitis—The Prospects for New Treatments. Nat. Rev. Neurol..

[54] Campbell G.L., Marfin A.A., Lanciotti R.S., Gubler D.J. (2002). West Nile Virus. Lancet Infect. Dis..

[55] Baer G.M. (2017). History of Rabies and Global Aspects.

[56] Sperk M., Van Domselaar R., Rodriguez J.E., Mikaeloff F., Sá Vinhas B., Saccon E., Sönnerborg A., Singh K., Gupta S., Végvári Á. (2020). Utility of Proteomics in Emerging and Re-Emerging Infectious Diseases Caused by RNA Viruses. J. Proteome Res..

[57] Çelik İ., Saatçi E., Eyüboğlu F.Ö. (2020). Emerging and Reemerging Respiratory Viral Infections up to COVID-19. Turk. J. Med. Sci..

[58] Curry S. (2015). Structural Biology: A Century-Long Journey into an Unseen World. Interdiscip. Sci. Rev..

[59] Brooks-Bartlett J.C., Garman E.F. (2015). The Nobel Science: One Hundred Years of Crystallography. Interdiscip. Sci. Rev..

[60] Thomas J.M. (2012). Centenary: The Birth of X-Ray Crystallography. Nature.

[61] Shi Y. (2014). A Glimpse of Structural Biology through X-Ray Crystallography. Cell.

[62] De Clercq E., Li G. (2016). Approved Antiviral Drugs over the Past 50 Years. Clin. Microbiol. Rev..

[63] Zheng H., Handing K.B., Zimmerman M.D., Shabalin I.G., Almo S.C., Minor W. (2015). X-Ray Crystallography over the Past Decade for Novel Drug Discovery—Where Are We Heading Next?. Expert Opin. Drug Discov..

[64] Stanley W.M. (1935). Isolation of a crystalline protein possessing the properties of tobacco-mosaic virus. Science.

[65] Norrby E. (2008). Nobel Prizes and the Emerging Virus Concept. Arch. Virol..

[66] Bernal J.D., Fankuchen I. (1941). X-Ray and crystallographic studies of plant virus preparations: I. introduction and preparation of specimens II. modes of aggregation of the virus particles. J. Gen. Physiol..

[67] Harrison S.C., Olson A.J., Schutt C.E., Winkler F.K., Bricogne G. (1978). Tomato Bushy Stunt Virus at 2.9 A Resolution. Nature.

[68] Bloomer A.C., Champness J.N., Bricogne G., Staden R., Klug A. (1978). Protein Disk of Tobacco Mosaic Virus at 2.8 A Resolution Showing the Interactions within and between Subunits. Nature.

[69] Richmond T.J., Finch J.T., Rushton B., Rhodes D., Klug A. (1984). Structure of the Nucleosome Core Particle at 7 A Resolution. Nature.

[70] Schirò A., Carlon A., Parigi G., Murshudov G., Calderone V., Ravera E., Luchinat C. (2020). On the Complementarity of X-Ray and NMR Data. J. Struct. Biol. X.

[71] Holcomb J., Spellmon N., Zhang Y., Doughan M., Li C., Yang Z. (2017). Protein Crystallization: Eluding the Bottleneck of X-Ray Crystallography. AIMS Biophys..

[72] Vincenzi M., Leone M. (2021). The Fight against Human Viruses: How NMR Can Help?. Curr. Med. Chem..

[73] Yu H. (1999). Extending the Size Limit of Protein Nuclear Magnetic Resonance. Proc. Natl. Acad. Sci. USA.

[74] LaPlante S.R., Coric P., Bouaziz S., França T.C.C. (2024). NMR Spectroscopy Can Help Accelerate Antiviral Drug Discovery Programs. Microbes Infect..

[75] Kruger D.H., Schneck P., Gelderblom H.R. (2000). Helmut Ruska and the Visualisation of Viruses. Lancet.

[76] Nagler F.P., Rake G. (1948). The Use of the Electron Microscope in Diagnosis of Variola, Vaccinia, and Varicella. J. Bacteriol..

[77] Brenner S., Horne R.W. (1959). A Negative Staining Method for High Resolution Electron Microscopy of Viruses. Biochim. Biophys. Acta.

[78] Tyrrell D.A.J., Almeida J.D. (1967). Direct Electron-Microscopy of Organ Cultures for the Detection and Characterization of Viruses. Arch. Gesamte Virusforsch..

[79] Adrian M., Dubochet J., Lepault J., McDowall A.W. (1984). Cryo-Electron Microscopy of Viruses. Nature.

[80] Schoehn G., Chenavier F., Crépin T. (2023). Advances in Structural Virology via Cryo-EM in 2022. Viruses.

[81] Dutta M., Acharya P. (2024). Cryo-Electron Microscopy in the Study of Virus Entry and Infection. Front. Mol. Biosci..

[82] Zhang X., Li S., Zhang K. (2024). Cryo-EM: A Window into the Dynamic World of RNA Molecules. Curr. Opin. Struct. Biol..

[83] Wu X., Rapoport T.A. (2021). Cryo-EM Structure Determination of Small Proteins by Nanobody-Binding Scaffolds (Legobodies). Proc. Natl. Acad. Sci. USA.

[84] Renaud J.P., Chari A., Ciferri C., Liu W.T., Rémigy H.W., Stark H., Wiesmann C. (2018). Cryo-EM in Drug Discovery: Achievements, Limitations and Prospects. Nat. Rev. Drug Discov..

[85] Boldon L., Laliberte F., Liu L. (2015). Review of the Fundamental Theories behind Small Angle X-Ray Scattering, Molecular Dynamics Simulations, and Relevant Integrated Application. Nano Rev..

[86] Handa T., Kundu D., Dubey V.K. (2023). Perspectives on Evolutionary and Functional Importance of Intrinsically Disordered Proteins. Int. J. Biol. Macromol..

[87] Barradas-Bautista D., Rosell M., Pallara C., Fernández-Recio J. (2018). Structural Prediction of Protein–Protein Interactions by Docking: Application to Biomedical Problems. Adv. Protein Chem. Struct. Biol..

[88] Zhu P., Winkler H., Chertova E., Taylor K.A., Roux K.H. (2008). Cryoelectron Tomography of HIV-1 Envelope Spikes: Further Evidence for Tripod-like Legs. PLoS Pathog..

[89] Baumeister W. (2022). Cryo-Electron Tomography: The Power of Seeing the Whole Picture. Biochem. Biophys. Res. Commun..

[90] Meents A., Wiedorn M.O. (2019). Virus Structures by X-Ray Free-Electron Lasers. Annu. Rev. Virol..

[91] Wang J. (2020). Fast Identification of Possible Drug Treatment of Coronavirus Disease-19 (COVID-19) through Computational Drug Repurposing Study. J. Chem. Inf. Model.

[92] Singh A., Dhaka P., Kumar P., Tomar S., Singla J. (2024). Bioinformatics Databases and Tools Available for the Development of Antiviral Drugs. Advances in Antiviral Research.

[93] Abramson J., Adler J., Dunger J., Evans R., Green T., Pritzel A., Ronneberger O., Willmore L., Ballard A.J., Bambrick J. (2024). Accurate Structure Prediction of Biomolecular Interactions with AlphaFold 3. Nature.

[94] O’Leary K. (2024). AlphaFold Gets an Upgrade (and a Nobel). Nat. Med..

[95] Fenner F., Bachmann P.A., Gibbs E.P.J., Murphy F.A., Studdert M.J., White D.O. (1987). Structure and Composition of Viruses. Veterinary Virology.

[96] Zheng B., Duan M., Huang Y., Wang S., Qiu J., Lu Z., Liu L., Tang G., Cheng L., Zheng P. (2024). Discovery of a Heparan Sulfate Binding Domain in Monkeypox Virus H3 as an Anti-Poxviral Drug Target Combining AI and MD Simulations. Elife.

[97] Delogu I., Pastorino B., Baronti C., Nougairède A., Bonnet E., de Lamballerie X. (2011). In Vitro Antiviral Activity of Arbidol against Chikungunya Virus and Characteristics of a Selected Resistant Mutant. Antivir. Res..

[98] Barrow E., Nicola A.V., Liu J. (2013). Multiscale Perspectives of Virus Entry via Endocytosis. Virol. J..

[99] Kim A.S., Diamond M.S. (2022). A Molecular Understanding of Alphavirus Entry and Antibody Protection. Nat. Rev. Microbiol..

[100] Melton J.V., Ewart G.D., Weir R.C., Board P.G., Lee E., Gage P.W. (2002). Alphavirus 6K Proteins Form Ion Channels. J. Biol. Chem..

[101] Button J.M., Mukhopadhyay S. (2021). Capsid-E2 Interactions Rescue Core Assembly in Viruses That Cannot Form Cytoplasmic Nucleocapsid Cores. J. Virol..

[102] Liu D.X., Liang J.Q., Fung T.S. (2021). Human Coronavirus-229E, -OC43, -NL63, and -HKU1 (Coronaviridae). Encyclopedia of Virology: Volume 1–5.

[103] Schlicksup C.J., Zlotnick A. (2020). Viral Structural Proteins as Targets for Antivirals. Curr. Opin. Virol..

[104] Chen N., Zhang B., Deng L., Liang B., Ping J. (2022). Virus-Host Interaction Networks as New Antiviral Drug Targets for IAV and SARS-CoV-2. Emerg. Microbes Infect..

[105] Burrell C.J., Howard C.R., Murphy F.A. (2017). Virion Structure and Composition. Fenner White’s Med. Virol..

[106] Kaur R., Neetu, Mudgal R., Jose J., Kumar P., Tomar S. (2019). Glycan-Dependent Chikungunya Viral Infection Divulged by Antiviral Activity of NAG Specific Chi-like Lectin. Virology.

[107] Kosik I., Yewdell J.W. (2019). Influenza Hemagglutinin and Neuraminidase: Yin–Yang Proteins Coevolving to Thwart Immunity. Viruses.

[108] Checkley M.A., Luttge B.G., Freed E.O. (2011). HIV-1 Envelope Glycoprotein Biosynthesis, Trafficking, and Incorporation. J. Mol. Biol..

[109] Wrobel A.G. (2023). Mechanism and Evolution of Human ACE2 Binding by SARS-CoV-2 Spike. Curr. Opin. Struct. Biol..

[110] Epand R.M. (2003). Fusion Peptides and the Mechanism of Viral Fusion. Biochim. Biophys. Acta (BBA) Biomembr..

[111] Zhai X., Yuan Y., He W.T., Wu Y., Shi Y., Su S., Du Q., Mao Y. (2024). Evolving Roles of Glycosylation in the Tug-of-War between Virus and Host. Natl. Sci. Rev..

[112] Matsuyama S., Taguchi F. (2009). Two-Step Conformational Changes in a Coronavirus Envelope Glycoprotein Mediated by Receptor Binding and Proteolysis. J. Virol..

[113] Marzinek J.K., Raghuvamsi Palur V., Salem G., Chen F.-C., Wu S.-R., Bond P.J., Chao D.-Y. (2023). Uncovering the Conformational Dynamics of Dengue Virus and Its Virus-like Particles as Novel Vaccine Candidates. Biophys. J..

[114] Chen F., Nagy K., Chavez D., Willis S., McBride R., Giang E., Honda A., Bukh J., Ordoukhanian P., Zhu J. (2020). Antibody Responses to Immunization With HCV Envelope Glycoproteins as a Baseline for B-Cell-Based Vaccine Development. Gastroenterology.

[115] Katze M.G., He Y., Gale M. (2002). Viruses and Interferon: A Fight for Supremacy. Nat. Rev. Immunol..

[116] Zhang S., Xue X., Qiao S., Jia L., Wen X., Wang Y., Wang C., Li H., Cui J. (2023). Umifenovir Epigenetically Targets the IL-10 Pathway in Therapy against Coxsackievirus B4 Infection. Microbiol. Spectr..

[117] Sargsyan K., Mazmanian K., Lim C. (2023). A Strategy for Evaluating Potential Antiviral Resistance to Small Molecule Drugs and Application to SARS-CoV-2. Sci. Rep..

[118] McCallum M., Czudnochowski N., Rosen L.E., Zepeda S.K., Bowen J.E., Walls A.C., Hauser K., Joshi A., Stewart C., Dillen J.R. (2022). Structural Basis of SARS-CoV-2 Omicron Immune Evasion and Receptor Engagement. Science.

[119] Tang H., Ke Y., Liao Y., Bian Y., Yuan Y., Wang Z., Yang L., Ma H., Sun T., Zhang B. (2022). Mutational Escape Prevention by Combination of Four Neutralizing Antibodies That Target RBD Conserved Regions and Stem Helix. Virol. Sin..

[120] Shih H.I., Wang Y.C., Wang Y.P., Chi C.Y., Chien Y.W. (2024). Risk of Severe Dengue during Secondary Infection: A Population-Based Cohort Study in Taiwan. J. Microbiol. Immunol. Infect..

[121] Wells T.J., Esposito T., Henderson I.R., Labzin L.I. (2024). Mechanisms of Antibody-Dependent Enhancement of Infectious Disease. Nat. Rev. Immunol..

[122] Sarker A., Dhama N., Gupta R.D. (2023). Dengue Virus Neutralizing Antibody: A Review of Targets, Cross-Reactivity, and Antibody-Dependent Enhancement. Front. Immunol..

[123] Malik S., Ahsan O., Mumtaz H., Tahir Khan M., Sah R., Waheed Y. (2023). Tracing down the Updates on Dengue Virus—Molecular Biology, Antivirals, and Vaccine Strategies. Vaccines.

[124] Dengvaxia|European Medicines Agency (EMA). https://www.ema.europa.eu/en/medicines/human/EPAR/dengvaxia.

[125] Ragonnet-Cronin M., Nutalai R., Huo J., Dijokaite-Guraliuc A., Das R., Tuekprakhon A., Supasa P., Liu C., Selvaraj M., Groves N. (2023). Generation of SARS-CoV-2 Escape Mutations by Monoclonal Antibody Therapy. Nat. Commun..

[126] Choudhary S., Malik Y.S., Tomar S. (2020). Identification of SARS-CoV-2 Cell Entry Inhibitors by Drug Repurposing Using in Silico Structure-Based Virtual Screening Approach. Front. Immunol..

[127] Hayden F.G., Osterhaus A.D.M.E., Treanor J.J., Fleming D.M., Aoki F.Y., Nicholson K.G., Bohnen A.M., Hirst H.M., Keene O., Wightman K. (1997). Efficacy and Safety of the Neuraminidase Inhibitor Zanamivir in the Treatment of Influenzavirus Infections. N. Engl. J. Med..

[128] Collins P.J., Haire L.F., Lin Y.P., Liu J., Russell R.J., Walker P.A., Skehel J.J., Martin S.R., Hay A.J., Gamblin S.J. (2008). Crystal Structures of Oseltamivir-Resistant Influenza Virus Neuraminidase Mutants. Nature.

[129] Vavricka C.J., Li Q., Wu Y., Qi J., Wang M., Liu Y., Gao F., Liu J., Feng E., He J. (2011). Structural and Functional Analysis of Laninamivir and Its Octanoate Prodrug Reveals Group Specific Mechanisms for Influenza NA Inhibition. PLoS Pathog..

[130] Pattnaik G.P., Chakraborty H. (2020). Entry Inhibitors: Efficient Means to Block Viral Infection. J. Membr. Biol..

[131] Beugeling M., De Zee J., Woerdenbag H.J., Frijlink H.W., Wilschut J.C., Hinrichs W.L.J. (2019). Respiratory Syncytial Virus Subunit Vaccines Based on the Viral Envelope Glycoproteins Intended for Pregnant Women and the Elderly. Expert Rev. Vaccines.

[132] Singh V.A., Nehul S., Kumar C.S., Banerjee M., Kumar P., Sharma G., Tomar S. (2023). Chimeric Chikungunya Virus-like Particles with Surface Exposed SARS-CoV-2 RBD Elicits Potent Immunogenic Responses in Mice. bioRxiv.

[133] Singh V.A., Kumar C.S., Khare B., Kuhn R.J., Banerjee M., Tomar S. (2023). Surface Decorated Reporter-Tagged Chikungunya Virus-like Particles for Clinical Diagnostics and Identification of Virus Entry Inhibitors. Virology.

[134] Nieva J.L., Madan V., Carrasco L. (2012). Viroporins: Structure and Biological Functions. Nat. Rev. Microbiol..

[135] Xia X., Cheng A., Wang M., Ou X., Sun D., Mao S., Huang J., Yang Q., Wu Y., Chen S. (2022). Functions of Viroporins in the Viral Life Cycle and Their Regulation of Host Cell Responses. Front. Immunol..

[136] Devantier K., Kjær V.M.S., Griffin S., Kragelund B.B., Rosenkilde M.M. (2024). Advancing the Field of Viroporins—Structure, Function and Pharmacology: IUPHAR Review 39. Br. J. Pharmacol..

[137] Pinto L.H., Holsinger L.J., Lamb R.A. (1992). Influenza Virus M2 Protein Has Ion Channel Activity. Cell.

[138] Surya W., Samsó M., Torres J. (2013). Structural and Functional Aspects of Viroporins in Human Respiratory Viruses: Respiratory Syncytial Virus and Coronaviruses. Respiratory Disease and Infection-A New Insight.

[139] Das K. (2012). Antivirals Targeting Influenza a Virus. J. Med. Chem..

[140] Thomaston J.L., Polizzi N.F., Konstantinidi A., Wang J., Kolocouris A., Degrado W.F. (2018). Inhibitors of the M2 Proton Channel Engage and Disrupt Transmembrane Networks of Hydrogen-Bonded Waters. J. Am. Chem. Soc..

[141] Nieto-Torres J.L., Verdiá-Báguena C., Castaño-Rodriguez C., Aguilella V.M., Enjuanes L. (2015). Relevance of Viroporin Ion Channel Activity on Viral Replication and Pathogenesis. Viruses.

[142] Dey D., Siddiqui S.I., Mamidi P., Ghosh S., Kumar C.S., Chattopadhyay S., Ghosh S., Banerjee M. (2019). The Effect of Amantadine on an Ion Channel Protein from Chikungunya Virus. PLoS Negl. Trop. Dis..

[143] Lamb R.A. (2008). Influenza. Encycl. Virol..

[144] Scott C., Griffin S. (2015). Viroporins: Structure, Function and Potential as Antiviral Targets. J. Gen. Virol..

[145] Fatma B., Kumar R., Singh V.A., Nehul S., Sharma R., Kesari P., Kuhn R.J., Tomar S. (2020). Alphavirus Capsid Protease Inhibitors as Potential Antiviral Agents for Chikungunya Infection. Antivir. Res..

[146] Chakravarty A., Rao A.L. (2021). The Interplay between Capsid Dynamics and Pathogenesis in Tripartite Bromoviruses. Curr. Opin. Virol..

[147] Ghaemi Z., Gruebele M., Tajkhorshid E. (2021). Molecular Mechanism of Capsid Disassembly in Hepatitis B Virus. Proc. Natl. Acad. Sci. USA.

[148] Mohajerani F., Tyukodi B., Schlicksup C.J., Hadden-Perilla J.A., Zlotnick A., Hagan M.F. (2022). Multiscale Modeling of Hepatitis B Virus Capsid Assembly and Its Dimorphism. ACS Nano.

[149] Koehl P., Akopyan A., Edelsbrunner H. (2023). Computing the Volume, Surface Area, Mean, and Gaussian Curvatures of Molecules and Their Derivatives. J. Chem. Inf. Model.

[150] Aggarwal M., Kaur R., Saha A., Mudgal R., Yadav R., Dash P.K., Parida M., Kumar P., Tomar S. (2017). Evaluation of Antiviral Activity of Piperazine against Chikungunya Virus Targeting Hydrophobic Pocket of Alphavirus Capsid Protein. Antivir. Res..

[151] Thenin-Houssier S., T Valente S. (2016). HIV-1 Capsid Inhibitors as Antiretroviral Agents. Curr. HIV Res..

[152] Segal-Maurer S., DeJesus E., Stellbrink H.-J., Castagna A., Richmond G.J., Sinclair G.I., Siripassorn K., Ruane P.J., Berhe M., Wang H. (2022). Capsid Inhibition with Lenacapavir in Multidrug-Resistant HIV-1 Infection. N. Engl. J. Med..

[153] Klumpp K., Crépin T. (2014). Capsid Proteins of Enveloped Viruses as Antiviral Drug Targets. Curr. Opin. Virol..

[154] Zhang X., Zhang Y., Jia R., Wang M., Yin Z., Cheng A. (2021). Structure and Function of Capsid Protein in Flavivirus Infection and Its Applications in the Development of Vaccines and Therapeutics. Vet. Res..

[155] Zhang X., Jia R., Zhou J., Wang M., Yin Z., Cheng A. (2016). Capsid-Targeted Viral Inactivation: A Novel Tactic for Inhibiting Replication in Viral Infections. Viruses.

[156] Dhaka P., Mahto J.K., Singh A., Kumar P., Tomar S. (2024). Structural Insights into the RNA Binding Inhibitors of the C-Terminal Domain of the SARS-CoV-2 Nucleocapsid. bioRxiv.

[157] Dhaka P., Singh A., Choudhary S., Peddinti R.K., Kumar P., Sharma G.K., Tomar S. (2023). Mechanistic and Thermodynamic Characterization of Antiviral Inhibitors Targeting Nucleocapsid N-Terminal Domain of SARS-CoV-2. Arch. Biochem. Biophys..

[158] Stanley W.M. (1946). The Isolation and Properties of Crystalline Tobacco Mosaic Virus. Nobel Lect..

[159] Namba K., Pattanayek R., Stubbs G. (1989). Visualization of Protein-Nucleic Acid Interactions in a Virus: Refined Structure of Intact Tobacco Mosaic Virus at 2.9 Å Resolution by X-Ray Fiber Diffraction. J. Mol. Biol..

[160] Aggarwal M., Tapas S., Preeti, Siwach A., Kumar P., Kuhn R.J., Tomar S. (2012). Crystal Structure of Aura Virus Capsid Protease and Its Complex with Dioxane: New Insights into Capsid-Glycoprotein Molecular Contacts. PLoS ONE.

[161] Aggarwal M., Dhindwal S., Kumar P., Kuhn R.J., Tomar S. (2014). Trans -Protease Activity and Structural Insights into the Active Form of the Alphavirus Capsid Protease. J. Virol..

[162] Kanodia S., Da Silva D.M., Kast W.M. (2008). Recent Advances in Strategies for Immunotherapy of Human Papillomavirus-Induced Lesions. Int. J. Cancer.

[163] Demmler-Harrison G.J. (2009). Antiviral Agents. Feigin and Cherry’s Textbook of Pediatric Infectious Diseases.

[164] Wlodawer A., Vondrasek J. (1998). Inhibitors of HIV-1 Protease: A Major Success of Structure-Assisted Drug Design. Annu. Rev. Biophys. Biomol. Struct..

[165] FDA-Approved HIV Medicines|NIH. https://hivinfo.nih.gov/understanding-hiv/fact-sheets/fda-approved-hiv-medicines.

[166] Weber I.T., Miller M., Jaskólski M., Leis J., Skalka A.M., Wlodawer A. (1989). Molecular Modeling of the HIV-1 Protease and Its Substrate Binding Site. Science.

[167] Lapatto R., Blundell T., Hemmings A., Overington J., Wilderspin A., Wood S., Merson J.R., Whittle P.J., Danley D.E., Geoghegan K.F. (1989). X-Ray Analysis of HIV-1 Proteinase at 2.7 Å Resolution Confirms Structural Homology among Retroviral Enzymes. Nature.

[168] Roberts N.A., Martin J.A., Kinchington D., Broadhurst A.V., Craig J.C., Duncan I.B., Galpin S.A., Handa B.K., Kay J., Kröhn A. (1990). Rational Design of Peptide-Based HIV Proteinase Inhibitors. Science (1979).

[169] Craig J.C., Duncan I.B., Hockley D., Grief C., Roberts N.A., Mills J.S. (1991). Antiviral Properties of Ro 31-8959, an Inhibitor of Human Immunodeficiency Virus (HIV) Proteinase. Antivir. Res..

[170] Kempf D.J., Marsh K.C., Denissen J.F., McDonald E., Vasavanonda S., Flentge C.A., Green B.E., Fino L., Park C.H., Kong X.P. (1995). ABT-538 Is a Potent Inhibitor of Human Immunodeficiency Virus Protease and Has High Oral Bioavailability in Humans. Proc. Natl. Acad. Sci. USA.

[171] Erickson J.W. (1993). Design and Structure of Symmetry-Based Inhibitors of HIV-1 Protease. Perspect. Drug Discov. Des..

[172] Dorsey B.D., Levin R.B., McDaniel S.L., Vacca J.P., Guare J.P., Anderson P.S., Huff J.R., Darke P.L., Zugay J.A., Emini E.A. (1994). L-735,524: The Design of a Potent and Orally Bioavailable HIV Protease Inhibitor. J. Med. Chem..

[173] Vacca J.P., Dorsey B.D., Schleif W.A., Levin R.B., Mcdaniel S.L., Darke P.L., Zugay J., Quintero J.C., Blahy O.M., Roth E. (1994). L-735,524: An Orally Bioavailable Human Immunodeficiency Virus Type 1 Protease Inhibitor. Proc. Natl. Acad. Sci. USA.

[174] Wlodawer A. (2002). Rational Approach to AIDS Drug Design through Structural Biology. Annu. Rev. Med..

[175] Varney M.D., Appelt K., Kalish V., Reddy M.R., Tatlock J., Palmer C.L., Romines W.H., Wu B.W., Musick L. (1994). Crystal-Structure-Based Design and Synthesis of Novel C-Terminal Inhibitors of HIV Protease. J. Med. Chem..

[176] Kaldor S.W., Kalish V.J., Davies J.F., Shetty B.V., Fritz J.E., Appelt K., Burgess J.A., Campanale K.M., Chirgadze N.Y., Clawson D.K. (1997). Viracept (Nelfinavir Mesylate, AG1343): A Potent, Orally Bioavailable Inhibitor of HIV-1 Protease. J. Med. Chem..

[177] Weber I.T., Waltman M.J., Mustyakimov M., Blakeley M.P., Keen D.A., Ghosh A.K., Langan P., Kovalevsky A.Y. (2013). Joint X-Ray/Neutron Crystallographic Study of HIV-1 Protease with Clinical Inhibitor Amprenavir: Insights for Drug Design. J. Med. Chem..

[178] Chemburkar S.R., Bauer J., Deming K., Spiwek H., Patel K., Morris J., Henry R., Spanton S., Dziki W., Porter W. (2000). Dealing with the Impact of Ritonavir Polymorphs on the Late Stages of Bulk Drug Process Development. Org. Process. Res. Dev..

[179] McCauley J.A., Rudd M.T. (2016). Hepatitis C Virus NS3/4a Protease Inhibitors. Curr. Opin. Pharmacol..

[180] Love R.A., Parge H.E., Wickersham J.A., Hostomsky Z., Habuka N., Moomaw E.W., Adachi T., Hostomska Z. (1996). The Crystal Structure of Hepatitis C Virus NS3 Proteinase Reveals a Trypsin-like Fold and a Structural Zinc Binding Site. Cell.

[181] Kim J.L., Morgenstern K.A., Lin C., Fox T., Dwyer M.D., Landro J.A., Chambers S.P., Markland W., Lepre C.A., O’Malley E.T. (1996). Crystal Structure of the Hepatitis C Virus NS3 Protease Domain Complexed with a Synthetic NS4A Cofactor Peptide. Cell.

[182] Barbato G., Cicero D.O., Nardi M.C., Steinkühler C., Cortese R., De Francesco R., Bazzo R. (1999). The Solution Structure of the N-Terminal Proteinase Domain of the Hepatitis C Virus (HCV) NS3 Protein Provides New Insights into Its Activation and Catalytic Mechanism. J. Mol. Biol..

[183] Llinàs-Brunet M., Bailey M., Fazal G., Goulet S., Halmos T., Laplante S., Maurice R., Poirier M., Poupart M.A., Thibeault D. (1998). Peptide-Based Inhibitors of the Hepatitis C Virus Serine Protease. Bioorg. Med. Chem. Lett..

[184] Saalau-Bethell S.M., Woodhead A.J., Chessari G., Carr M.G., Coyle J., Graham B., Hiscock S.D., Murray C.W., Pathuri P., Rich S.J. (2012). Discovery of an Allosteric Mechanism for the Regulation of HcV Ns3 Protein Function. Nat. Chem. Biol..

[185] Choudhary S., Nehul S., Singh A., Panda P.K., Kumar P., Sharma G.K., Tomar S. (2024). Unraveling Antiviral Efficacy of Multifunctional Immunomodulatory Triterpenoids against SARS-COV-2 Targeting Main Protease and Papain-like Protease. IUBMB Life.

[186] Choudhary S., Nehul S., Nagaraj S.K., Narayan R., Verma S., Sharma S., Kumari A., Rani R., Saha A., Sircar D. (2022). Activity Profiling of Deubiquitinating Inhibitors-Bound to SARS-CoV-2 Papain like Protease with Antiviral Efficacy in Murine Infection Model. bioRxiv.

[187] Owen D.R., Allerton C.M.N., Anderson A.S., Aschenbrenner L., Avery M., Berritt S., Boras B., Cardin R.D., Carlo A., Coffman K.J. (2021). An Oral SARS-CoV-2 Mpro Inhibitor Clinical Candidate for the Treatment of COVID-19. Science.

[188] Zhang L., Lin D., Sun X., Curth U., Drosten C., Sauerhering L., Becker S., Rox K., Hilgenfeld R. (2020). Crystal Structure of SARS-CoV-2 Main Protease Provides a Basis for Design of Improved a-Ketoamide Inhibitors. Science.

[189] FDA Approves First Oral Antiviral for Treatment of COVID-19 in Adults|FDA. https://www.fda.gov/news-events/press-announcements/fda-approves-first-oral-antiviral-treatment-covid-19-adults.

[190] Pagliano P., Spera A., Sellitto C., Scarpati G., Folliero V., Piazza O., Franci G., Conti V., Ascione T. (2023). Preclinical Discovery and Development of Nirmatrelvir/Ritonavir Combinational Therapy for the Treatment of COVID-19 and the Lessons Learned from SARS-COV-2 Variants. Expert Opin. Drug Discov..

[191] Unoh Y., Uehara S., Nakahara K., Nobori H., Yamatsu Y., Yamamoto S., Maruyama Y., Taoda Y., Kasamatsu K., Suto T. (2022). Discovery of S-217622, a Noncovalent Oral SARS-CoV-2 3CL Protease Inhibitor Clinical Candidate for Treating COVID-19. J. Med. Chem..

[192] Narwal M., Armache J.P., Edwards T.J., Murakami K.S. (2023). SARS-CoV-2 Polyprotein Substrate Regulates the Stepwise Mpro Cleavage Reaction. J. Biol. Chem..

[193] Pareek A., Kumar R., Mudgal R., Neetu N., Sharma M., Kumar P., Tomar S. (2022). Alphavirus Antivirals Targeting RNA-Dependent RNA Polymerase Domain of NsP4 Divulged Using Surface Plasmon Resonance. FEBS J..

[194] Rani R., Long S., Pareek A., Dhaka P., Singh A., Kumar P., McInerney G., Tomar S. (2022). Multi-Target Direct-Acting SARS-CoV-2 Antivirals against the Nucleotide-Binding Pockets of Virus-Specific Proteins. Virology.

[195] Rani R., Nehul S., Choudhary S., Upadhyay A., Kumar Sharma G., Kumar P., Tomar S., scientist S. (2023). Revealing and Evaluation of Antivirals Targeting Multiple Druggable Sites of RdRp Complex in SARS-CoV-2. bioRxiv.

[196] Choi K.H. (2012). Viral Polymerases. Adv. Exp. Med. Biol..

[197] Liu S., Knafels J.D., Chang J.S., Waszak G.A., Baldwin E.T., Deibel M.R., Thomsen D.R., Homa F.L., Wells P.A., Tory M.C. (2006). Crystal Structure of the Herpes Simplex Virus 1 DNA Polymerase. J. Biol. Chem..

[198] Zarrouk K., Piret J., Boivin G. (2017). Herpesvirus DNA Polymerases: Structures, Functions and Inhibitors. Virus Res..

[199] Gustavsson E., Grünewald K., Elias P., Hällberg B.M. (2024). Dynamics of the Herpes Simplex Virus DNA Polymerase Holoenzyme during DNA Synthesis and Proof-Reading Revealed by Cryo-EM. Nucleic Acids Res..

[200] Shankar S., Pan J., Yang P., Bian Y., Oroszlán G., Yu Z., Mukherjee P., Filman D.J., Hogle J.M., Shekhar M. (2024). Viral DNA Polymerase Structures Reveal Mechanisms of Antiviral Drug Resistance. Cell.

[201] D’Cruz O.J., Uckun F.M. (2006). Dawn of Non-Nucleoside Inhibitor-Based Anti-HIV Microbicides. J. Antimicrob. Chemother..

[202] Tuaillon E., Gueudin M., Lemée V., Gueit I., Roques P., Corrigan G.E., Plantier J.C., Simon F., Braun J. (2024). Phenotypic susceptibility to nonnucleoside inhibitors of virion-associated reverse transcriptase from different HIV types and groups. JAIDS J. Acquir. Immune Defic. Syndr..

[203] Smerdon S.J., Jäger J., Wang J., Kohlstaedt L.A., Chirino A.J., Friedman J.M., Rice P.A., Steitz T.A. (1994). Structure of the Binding Site for Nonnucleoside Inhibitors of the Reverse Transcriptase of Human Immunodeficiency Virus Type 1. Proc. Natl. Acad. Sci. USA.

[204] Merluzzi V.J., Hargrave K.D., Labadia M., Grozinger K., Skoog M., Wu J.C., Shih C.-K., Eckner K., Hattox S., Adams J. (1990). Inhibition of HIV-1 Replication by a Nonnucleoside Reverse Transcriptase Inhibitor. Science.

[205] Omoto S., Speranzini V., Hashimoto T., Noshi T., Yamaguchi H., Kawai M., Kawaguchi K., Uehara T., Shishido T., Naito A. (2018). Characterization of Influenza Virus Variants Induced by Treatment with the Endonuclease Inhibitor Baloxavir Marboxil. Sci. Rep..

[206] Chang S., Sun D., Liang H., Wang J., Li J., Guo L., Wang X., Guan C., Boruah B.M., Yuan L. (2015). Cryo-EM Structure of Influenza Virus RNA Polymerase Complex at 4.3Å Resolution. Mol. Cell.

[207] Kirchdoerfer R.N., Ward A.B. (2019). Structure of the SARS-CoV Nsp12 Polymerase Bound to Nsp7 and Nsp8 Co-Factors. Nat. Commun..

[208] FDA Approves First Treatment for COVID-19|FDA. https://www.fda.gov/news-events/press-announcements/fda-approves-first-treatment-covid-19.

[209] Yin W., Mao C., Luan X., Shen D.D., Shen Q., Su H., Wang X., Zhou F., Zhao W., Gao M. (2020). Structural Basis for Inhibition of the RNA-Dependent RNA Polymerase from SARS-CoV-2 by Remdesivir. Science.

[210] Elfiky A.A. (2020). Ribavirin, Remdesivir, Sofosbuvir, Galidesivir, and Tenofovir against SARS-CoV-2 RNA Dependent RNA Polymerase (RdRp): A Molecular Docking Study. Life Sci..

[211] Sangawa H., Komeno T., Nishikawa H., Yoshida A., Takahashi K., Nomura N., Furuta Y. (2013). Mechanism of Action of T-705 Ribosyl Triphosphate against Influenza Virus RNA Polymerase. Antimicrob. Agents Chemother..

[212] Painter G.R., Natchus M.G., Cohen O., Holman W., Painter W.P. (2021). Developing a Direct Acting, Orally Available Antiviral Agent in a Pandemic: The Evolution of Molnupiravir as a Potential Treatment for COVID-19. Curr. Opin. Virol..

[213] Yoon J.J., Toots M., Lee S., Lee M.E., Ludeke B., Luczo J.M., Ganti K., Cox R.M., Sticher Z.M., Edpuganti V. (2018). Orally Efficacious Broad-Spectrum Ribonucleoside Analog Inhibitor of Influenza and Respiratory Syncytial Viruses. Antimicrob. Agents Chemother..

[214] Peng Q., Peng R., Yuan B., Wang M., Zhao J., Fu L., Qi J., Shi Y. (2021). Structural Basis of SARS-CoV-2 Polymerase Inhibition by Favipiravir. Innovation.

[215] Pommier Y., Johnson A.A., Marchand C. (2005). Integrase Inhibitors to Treat HIV/AIDS. Nat. Rev. Drug Discov..

[216] Cai M., Zheng R., Caffrey M., Craigie R., Marius Clore G., Gronenborn A.M. (1997). Solution Structure of the N-Terminal Zinc Binding Domain of HIV-1 Integrase. Nat. Struct. Bio.l.

[217] Renzi G., Carta F., Supuran C.T. (2023). The Integrase: An Overview of a Key Player Enzyme in the Antiviral Scenario. Int. J. Mol. Sci..

[218] Maertens G.N., Engelman A.N., Cherepanov P. (2022). Structure and Function of Retroviral Integrase. Nat Rev Microbiol..

[219] Blanco J.L., Whitlock G., Milinkovic A., Moyle G. (2015). HIV Integrase Inhibitors: A New Era in the Treatment of HIV. Expert Opin. Pharmacother..

[220] Goldgur Y., Craigie R., Cohen G.H., Fujiwara T., Yoshinaga T., Fujishita T., Sugimoto H., Endo T., Murai H., Davies D.R. (1999). Structure of the HIV-1 Integrase Catalytic Domain Complexed with an Inhibitor: A Platform for Antiviral Drug Design. Proc. Natl. Acad. Sci. USA.

[221] Hare S., Smith S.J., Métifiot M., Jaxa-Chamiec A., Pommier Y., Hughes S.H., Cherepanov P. (2011). Structural and Functional Analyses of the Second-Generation Integrase Strand Transfer Inhibitor Dolutegravir (S/GSK1349572). Mol. Pharmacol..

[222] Ballantyne A.D., Perry C.M. (2013). Dolutegravir: First Global Approval. Drugs.

[223] New report documents increase in HIV drug resistance to dolutegravir|WHO. https://www.who.int/news/item/05-03-2024-new-report-documents-increase-in-hiv-drug-resistance-to-dolutegravir.

[224] Li M., Passos D.O., Shan Z., Smith S.J., Sun Q., Biswas A., Choudhuri I., Strutzenberg T.S., Haldane A., Deng N. (2023). Mechanisms of HIV-1 Integrase Resistance to Dolutegravir and Potent Inhibition of Drug-Resistant Variants. Sci. Adv..

[225] Passos D.O., Li M., Jóźwik I.K., Zhao X.Z., Santos-Martins D., Yang R., Smith S.J., Jeon Y., Forli S., Hughes S.H. (2020). Structural Basis for Strand-Transfer Inhibitor Binding to HIV Intasomes. Science.

[226] Bonnard D., Le Rouzic E., Eiler S., Amadori C., Orlov I., Bruneau J.M., Brias J., Barbion J., Chevreuil F., Spehner D. (2018). Structure-Function Analyses Unravel Distinct Effects of Allosteric Inhibitors of HIV-1 Integrase on Viral Maturation and Integration. J. Biol. Chem..

[227] Christ F., Voet A., Marchand A., Nicolet S., Desimmie B.A., Marchand D., Bardiot D., Van Der Veken N.J., Van Remoortel B., Strelkov S.V. (2010). Rational Design of Small-Molecule Inhibitors of the LEDGF/P75-Integrase Interaction and HIV Replication. Nat. Chem. Biol..

[228] Xie Y., Wu L., Wang M., Cheng A., Yang Q., Wu Y., Jia R., Zhu D., Zhao X., Chen S. (2019). Alpha-Herpesvirus Thymidine Kinase Genes Mediate Viral Virulence and Are Potential Therapeutic Targets. Front. Microbiol..

[229] Brown D.G., Visse R., Sandhu G., Davies A., Rizkallah P.J., Melitz C., Summers W.C., Sanderson M.R. (1995). Crystal Structures of the Thymidine Kinase from Herpes Simplex Virus Type-I in Complex with Deoxythymidine and Ganciclovir. Nat. Struct. Biol..

[230] Wild K., Bohner T., Aubry A., Folkers G., Schulz G.E. (1995). The Three-Dimensional Structure of Thymidine Kinase from Herpes Simplex Virus Type 1. FEBS Lett..

[231] Frobert E., Ooka T., Cortay J.C., Lina B., Thouvenot D., Morfin F. (2005). Herpes Simplex Virus Thymidine Kinase Mutations Associated with Resistance to Acyclovir: A Site-Directed Mutagenesis Study. Antimicrob. Agents Chemother..

[232] Ramdhan P., Li C. (2022). Targeting Viral Methyltransferases: An Approach to Antiviral Treatment for SsRNA Viruses. Viruses.

[233] Jones R., Bragagnolo G., Arranz R., Reguera J. (2021). Capping Pores of Alphavirus NsP1 Gate Membranous Viral Replication Factories. Nature.

[234] Lampio A., Kilpeläinen I., Pesonen S., Karhi K., Auvinen P., Somerharju P., Kääriäinen L. (2000). Membrane Binding Mechanism of an RNA Virus-Capping Enzyme. J. Biol. Chem..

[235] Jones R., Hons M., Rabah N., Zamarreño N., Arranz R., Reguera J. (2023). Structural Basis and Dynamics of Chikungunya Alphavirus RNA Capping by NsP1 Capping Pores. Proc. Natl. Acad. Sci. USA.

[236] Bhutkar M., Saha A., Tomar S. (2024). Viral Methyltransferase Inhibitors: Berbamine, Venetoclax, and Ponatinib as Efficacious Antivirals against Chikungunya Virus. Arch. Biochem. Biophys..

[237] Mudgal R., Bharadwaj C., Dubey A., Choudhary S., Nagarajan P., Aggarwal M., Ratra Y., Basak S., Tomar S. (2022). Selective Estrogen Receptor Modulators Limit Alphavirus Infection by Targeting the Viral Capping Enzyme NsP1. Antimicrob. Agents Chemother..

[238] Mudgal R., Mahajan S., Tomar S. (2020). Inhibition of Chikungunya Virus by an Adenosine Analog Targeting the SAM-Dependent NsP1 Methyltransferase. FEBS Lett..

[239] Zhou Y., Ray D., Zhao Y., Dong H., Ren S., Li Z., Guo Y., Bernard K.A., Shi P.-Y., Li H. (2007). Structure and Function of Flavivirus NS5 Methyltransferase. J. Virol..

[240] Lim S.P., Sonntag L.S., Noble C., Nilar S.H., Ng R.H., Zou G., Monaghan P., Chung K.Y., Dong H., Liu B. (2011). Small Molecule Inhibitors That Selectively Block Dengue Virus Methyltransferase. J. Biol. Chem..

[241] Upadhyay A.K., Cyr M., Longenecker K., Tripathi R., Sun C., Kempf D.J. (2017). Crystal Structure of Full-Length Zika Virus NS5 Protein Reveals a Conformation Similar to Japanese Encephalitis Virus NS5. Acta Crystallogr. Sect. F Struct. Biol. Commun..

[242] Jia H., Zhong Y., Peng C., Gong P. (2022). Crystal Structures of Flavivirus NS5 Guanylyltransferase Reveal a GMP-Arginine Adduct. J. Virol..

[243] Chen H., Lin S., Yang F., Chen Z., Guo L., Yang J., Lin X., Wang L., Duan Y., Wen A. (2023). Structural and Functional Basis of Low-Affinity SAM/SAH-Binding in the Conserved MTase of the Multi-Segmented Alongshan Virus Distantly Related to Canonical Unsegmented Flaviviruses. PLoS Pathog..

[244] Bhutkar M., Kumar A., Rani R., Singh V., Pathak A., Kothiala A., Mahajan S., Waghmode B., Kumar R., Mudgal R. (2024). SAM-Dependent Viral MTase Inhibitors: Herbacetin and Caffeic Acid Phenethyl Ester, Structural Insights into Dengue MTase. bioRxiv.

[245] García L.L., Padilla L., Castaño J.C. (2017). Inhibitors Compounds of the Flavivirus Replication Process. Virol. J..

[246] Benarroch D., Egloff M.P., Mulard L., Guerreiro C., Romette J.L., Canard B. (2004). A Structural Basis for the Inhibition of the NS5 Dengue Virus MRNA 2′-O-Methyltransferase Domain by Ribavirin 5′-Triphosphate. J. Biol. Chem..

[247] Milani M., Mastrangelo E., Bollati M., Selisko B., Decroly E., Bouvet M., Canard B., Bolognesi M. (2009). Flaviviral Methyltransferase/RNA Interaction: Structural Basis for Enzyme Inhibition. Antivir. Res..

[248] Nencka R., Silhan J., Klima M., Otava T., Kocek H., Krafcikova P., Boura E. (2022). Coronaviral RNA-Methyltransferases: Function, Structure and Inhibition. Nucleic Acids Res..

[249] Li X., Song Y. (2024). Perspective for Drug Discovery Targeting SARS Coronavirus Methyltransferases: Function, Structure and Inhibition. J. Med. Chem..

[250] Pyle A.M. (2008). Translocation and Unwinding Mechanisms of RNA and DNA Helicases. Annu. Rev. Biophys..

[251] Kolykhalov A.A., Mihalik K., Feinstone S.M., Rice C.M. (2000). Hepatitis C Virus-Encoded Enzymatic Activities and Conserved RNA Elements in the 3′ Nontranslated Region Are Essential for Virus Replication In Vivo. J. Virol..

[252] Jankowsky E., Gross C.H., Shuman S., Pyle A.M. (2000). The DExH Protein NPH-II Is a Processive and Directional Motor for Unwinding RNA. Nature.

[253] Newman J.A., Douangamath A., Yadzani S., Yosaatmadja Y., Aimon A., Brandão-Neto J., Dunnett L., Gorrie-stone T., Skyner R., Fearon D. (2021). Structure, Mechanism and Crystallographic Fragment Screening of the SARS-CoV-2 NSP13 Helicase. Nat. Commun..

[254] Smelkova N.V., Borowiec J.A. (1997). Dimerization of Simian Virus 40 T-Antigen Hexamers Activates T-Antigen DNA Helicase Activity. J. Virol..

[255] Hughes F.J., Romanos M.A. (1993). E1 Protein of Human Papillomavirus Is a DNA Helicase/ATPase. Nucleic Acids Res..

[256] Xu T., Sampath A., Chao A., Wen D., Nanao M., Chene P., Vasudevan S.G., Lescar J. (2005). Structure of the Dengue Virus Helicase/Nucleoside Triphosphatase Catalytic Domain at a Resolution of 2.4 Å. J. Virol..

[257] Zhang W., Liu Y., Yang M., Yang J., Shao Z., Gao Y., Jiang X., Cui R., Zhang Y., Zhao X. (2024). Structural and Functional Insights into the Helicase Protein E5 of Mpox Virus. Cell Discov..

[258] Law Y.-S., Wang S., Tan Y.B., Shih O., Utt A., Goh W.Y., Lian B.-J., Chen M.W., Jeng U.-S., Merits A. (2021). Interdomain Flexibility of Chikungunya Virus NsP2 Helicase-Protease Differentially Influences Viral RNA Replication and Infectivity. J. Virol..

[259] Lou Z., Sun Y., Rao Z. (2014). Current Progress in Antiviral Strategies. Trends Pharmacol. Sci..

[260] Wu J., Bera A.K., Kuhn R.J., Smith J.L. (2005). Structure of the Flavivirus Helicase: Implications for Catalytic Activity, Protein Interactions, and Proteolytic Processing. J. Virol..

[261] Tortorici M.A., Duquerroy S., Kwok J., Vonrhein C., Perez J., Lamp B., Bricogne G., Rümenapf T., Vachette P., Rey F.A. (2015). X-Ray Structure of the Pestivirus NS3 Helicase and Its Conformation in Solution. J. Virol..

[262] Fang X., Lu G., Deng Y., Yang S., Hou C., Gong P. (2023). Unusual Substructure Conformations Observed in Crystal Structures of a Dicistrovirus RNA-Dependent RNA Polymerase Suggest Contribution of the N-Terminal Extension in Proper Folding. Virol. Sin..

[263] Anindita P.D., Halbeisen M., Řeha D., Tuma R., Franta Z. (2022). Mechanistic Insight into the RNA-Stimulated ATPase Activity of Tick-Borne Encephalitis Virus Helicase. J. Biol. Chem..

[264] Shao Z., Su S., Yang J., Zhang W., Gao Y., Zhao X., Zhang Y., Shao Q., Cao C., Li H. (2023). Structures and Implications of the C962R Protein of African Swine Fever Virus. Nucleic Acids Res..

[265] Gu M., Rice C.M. (2010). Three Conformational Snapshots of the Hepatitis C Virus NS3 Helicase Reveal a Ratchet Translocation Mechanism. Proc. Natl. Acad. Sci. USA.

[266] Hutin S., Ling W.L., Tarbouriech N., Schoehn G., Grimm C., Fischer U., Burmeister W.P. (2022). The Vaccinia Virus DNA Helicase Structure from Combined Single-Particle Cryo-Electron Microscopy and AlphaFold2 Prediction. Viruses.

[267] Liu D., Wang Y.S., Gesell J.J., Wyss D.F. (2001). Solution Structure and Backbone Dynamics of an Engineered Arginine-Rich Subdomain 2 of the Hepatitis C Virus NS3 RNA Helicase. J. Mol. Biol..

[268] Frick D., Lam A. (2006). Understanding Helicases as a Means of Virus Control. Curr. Pharm. Des..

[269] Tan Q., Zhu Y., Li J., Chen Z., Han G.W., Kufareva I., Li T., Ma L., Fenalti G., Li J. (2013). Structure of the CCR5 Chemokine Receptor-HIV Entry Inhibitor Maraviroc Complex. Science.

[270] Wu B., Chien E.Y.T., Mol C.D., Fenalti G., Liu W., Katritch V., Abagyan R., Brooun A., Wells P., Bi F.C. (2010). Structures of the CXCR4 Chemokine GPCR with Small-Molecule and Cyclic Peptide Antagonists. Science.

[271] Haqqani A.A., Tilton J.C. (2013). Entry Inhibitors and Their Use in the Treatment of HIV-1 Infection. Antivir. Res..

[272] Blair H.A. (2020). Ibalizumab: A Review in Multidrug-Resistant HIV-1 Infection. Drugs.

[273] Freeman M.M., Seaman M.S., Rits-Volloch S., Hong X., Kao C.Y., Ho D.D., Chen B. (2010). Crystal Structure of HIV-1 Primary Receptor CD4 in Complex with a Potent Antiviral Antibody. Structure.

[274] Bhutkar M., Singh V., Dhaka P., Tomar S. (2022). Virus-Host Protein-Protein Interactions as Molecular Drug Targets for Arboviral Infections. Front. Virol..

[275] Mahajan S., Choudhary S., Kumar P., Tomar S. (2021). Antiviral Strategies Targeting Host Factors and Mechanisms Obliging +ssRNA Viral Pathogens. Bioorg. Med. Chem..

[276] Dhaka P., Singh A., Nehul S., Choudhary S., Panda P.K., Sharma G.K., Kumar P., Tomar S. (2024). Disruption of Molecular Interactions between the G3BP1 Stress Granule Host Protein and the Nucleocapsid (NTD-N) Protein Impedes SARS-CoV-2 Virus Replication. Biochemistry.

[277] Mahajan S., Kumar R., Singh A., Pareek A., Kumar P., Tomar S. (2024). Targeting the Host Protein G3BP1 for the Discovery of Novel Antiviral Inhibitors against Chikungunya Virus. bioRxiv.

[278] De Chassey B., Meyniel-Schicklin L., Aublin-Gex A., André P., Lotteau V. (2012). New Horizons for Antiviral Drug Discovery from Virus–Host Protein Interaction Networks. Curr. Opin. Virol..

[279] Idrees S., Chen H., Panth N., Paudel K.R., Hansbro P.M. (2024). Exploring Viral–Host Protein Interactions as Antiviral Therapies: A Computational Perspective. Microorganisms.

[280] Idrees S., Paudel K.R., Sadaf T., Hansbro P.M. (2023). How Different Viruses Perturb Host Cellular Machinery via Short Linear Motifs. EXCLI J..

[281] de Chassey B., Meyniel-Schicklin L., Vonderscher J., André P., Lotteau V. (2014). Virus-Host Interactomics: New Insights and Opportunities for Antiviral Drug Discovery. Genome Med..

[282] Brito A.F., Pinney J.W. (2017). Protein-Protein Interactions in Virus-Host Systems. Front. Microbiol..

[283] Kumar N., Sharma S., Kumar R., Tripathi B.N., Barua S., Ly H., Rouse B.T. (2020). Host-Directed Antiviral Therapy. Clin. Microbiol. Rev..

[284] Eslami N., Aghbash P.S., Shamekh A., Entezari-Maleki T., Nahand J.S., Sales A.J., Baghi H.B. (2022). SARS-CoV-2: Receptor and Co-Receptor Tropism Probability. Curr. Microbiol..

[285] Baggen J., Vanstreels E., Jansen S., Daelemans D. (2021). Cellular Host Factors for SARS-CoV-2 Infection. Nat. Microbiol..

[286] Lee M.F., Wu Y.S., Poh C.L. (2023). Molecular Mechanisms of Antiviral Agents against Dengue Virus. Viruses.

[287] Anwar M.N., Akhtar R., Abid M., Khan S.A., Rehman Z.U., Tayyub M., Malik M.I., Shahzad M.K., Mubeen H., Qadir M.S. (2022). The Interactions of Flaviviruses with Cellular Receptors: Implications for Virus Entry. Virology.

[288] Li H., Huang Q.Z., Zhang H., Liu Z.X., Chen X.H., Ye L.L., Luo Y. (2023). The Land-Scape of Immune Response to Monkeypox Virus. EBioMedicine.

[289] Chakraborty C., Sharma A.R., Bhattacharya M., Lee S.S. (2022). A Detailed Overview of Immune Escape, Antibody Escape, Partial Vaccine Escape of SARS-CoV-2 and Their Emerging Variants With Escape Mutations. Front. Immunol..

[290] Lemaitre J.C., Grantz K.H., Kaminsky J., Meredith H.R., Truelove S.A., Lauer S.A., Keegan L.T., Shah S., Wills J., Kaminsky K. (2021). A Scenario Modeling Pipeline for COVID-19 Emergency Planning. Sci. Rep..

[291] Crucitti D., Pérez Míguez C., Ángel J., Arias D., Beltrá D., Prada F., Orgueira A.M. (2024). De Novo Drug Design through Artificial Intelligence: An Introduction. Front. Hematol..

[292] Floresta G., Zagni C., Patamia V., Rescifina A. (2023). How Can Artificial Intelligence Be Utilized for de Novo Drug Design against COVID-19 (SARS-CoV-2)?. Expert Opin. Drug Discov..

[293] Patel C.N., Mall R., Bensmail H. (2023). AI-Driven Drug Repurposing and Binding Pose Meta Dynamics Identifies Novel Targets for Monkeypox Virus. J. Infect. Public Health.

[294] Slater A., Nair N., Suétt R., Mac Donnchadha R., Bamford C., Jasim S., Livingstone D., Hutchinson E. (2022). Visualising Viruses. J. Gen. Virol..

[295] Lu Y., Yang Q., Ran T., Zhang G., Li W., Zhou P., Tang J., Dai M., Zhong J., Chen H. (2024). Discovery of Orally Bioavailable SARS-CoV-2 Papain-like Protease Inhibitor as a Potential Treatment for COVID-19. Nat. Commun..

[296] Turzynski V., Monsees I., Moraru C., Probst A.J. (2021). Imaging Techniques for Detecting Prokaryotic Viruses in Environmental Samples. Viruses.

[297] Rut W., Lv Z., Zmudzinski M., Patchett S., Nayak D., Snipas S.J., El Oualid F., Huang T.T., Bekes M., Drag M. (2020). Activity Profiling and Crystal Structures of Inhibitor-Bound SARS-CoV-2 Papain-like Protease: A Framework for Anti–COVID-19 Drug Design. Sci. Adv..

[298] Greasley S.E., Noell S., Plotnikova O., Ferre R.A., Liu W., Bolanos B., Fennell K., Nicki J., Craig T., Zhu Y. (2022). Structural Basis for the in Vitro Efficacy of Nirmatrelvir against SARS-CoV-2 Variants. J. Biol. Chem..

[299] Feng Q., Cheng K., Zhang L., Wang D., Gao X., Liang J., Liu G., Ma N., Xu C., Tang M. (2024). Rationally Designed Multimeric Nanovaccines Using Icosahedral DNA Origami for Display of SARS-CoV-2 Receptor Binding Domain. Nat. Commun..

[300] Dokhale S., Garse S., Devarajan S., Thakur V., Kolhapure S. (2025). Rational Design of Antiviral Therapeutics. Comput. Methods Ration. Drug Des..

[301] Al-Amran F.G., Hezam A.M., Rawaf S., Yousif M.G. (2023). Genomic Analysis and Artificial Intelligence: Predicting Viral Mutations and Future Pandemics. arXiv.

[302] Parvatikar P.P., Patil S., Khaparkhuntikar K., Patil S., Singh P.K., Sahana R., Kulkarni R.V., Raghu A.V. (2023). Artificial Intelligence: Machine Learning Approach for Screening Large Database and Drug Discovery. Antivir. Res.

[303] KP Jayatunga M., Ayers M., Bruens L., Jayanth D., Meier C. (2024). How Successful Are AI-Discovered Drugs in Clinical Trials? A First Analysis and Emerging Lessons. Drug. Discov. Today.

[304] Mouchlis V.D., Afantitis A., Serra A., Fratello M., Papadiamantis A.G., Aidinis V., Lynch I., Greco D., Melagraki G. (2021). Advances in De Novo Drug Design: From Conventional to Machine Learning Methods. Int. J. Mol. Sci..

[305] Atz K., Cotos L., Isert C., Håkansson M., Focht D., Hilleke M., Nippa D.F., Iff M., Ledergerber J., Schiebroek C.C.G. (2024). Prospective de Novo Drug Design with Deep Interactome Learning. Nat. Commun..

[306] Singh V., Bhutkar M., Choudhary S., Nehul S., Kumar R., Singla J., Kumar P., Tomar S. (2024). Structure-Guided Mutations in CDRs for Enhancing the Affinity of Neutralizing SARS-CoV-2 Nanobody. Biochem. Biophys. Res. Commun..

[307] Singh V., Choudhary S., Bhutkar M., Nehul S., Ali S., Singla J., Kumar P., Tomar S. (2025). Designing and Bioengineering of CDRs with Higher Affinity against Receptor-Binding Domain (RBD) of SARS-CoV-2 Omicron Variant 2024. Int. J. Biol. Macromol..

[308] Sinha S., Vegesna R., Mukherjee S., Kammula A.V., Dhruba S.R., Wu W., Kerr D.L., Nair N.U., Jones M.G., Yosef N. (2024). PERCEPTION Predicts Patient Response and Resistance to Treatment Using Single-Cell Transcriptomics of Their Tumors. Nature Cancer.

[309] Mak K.-K., Wong Y.-H., Pichika M.R. (2024). Artificial Intelligence in Drug Discovery and Development. Drug Discovery and Evaluation: Safety and Pharmacokinetic Assays.

[310] He H., He B., Guan L., Zhao Y., Jiang F., Chen G., Zhu Q., Chen C.Y.C., Li T., Yao J. (2024). De Novo Generation of SARS-CoV-2 Antibody CDRH3 with a Pre-Trained Generative Large Language Model. Nat. Commun..

[311] Dunbar J., Krawczyk K., Leem J., Marks C., Nowak J., Regep C., Georges G., Kelm S., Popovic B., Deane C.M. (2016). SAbPred: A Structure-Based Antibody Prediction Server. Nucleic Acids Res..

[312] Caradonna T.M., Schmidt A.G. (2021). Protein Engineering Strategies for Rational Immunogen Design. npj Vaccines.

[313] Marcandalli J., Fiala B., Ols S., Perotti M., de van der Schueren W., Snijder J., Hodge E., Benhaim M., Ravichandran R., Carter L. (2019). Induction of Potent Neutralizing Antibody Responses by a Designed Protein Nanoparticle Vaccine for Respiratory Syncytial Virus. Cell.

[314] Shanehsazzadeh A., McPartlon M., Kasun G., Steiger A.K., Sutton J.M., Yassine E., McCloskey C., Haile R., Shuai R., Alverio J. (2024). Unlocking de Novo Antibody Design with Generative Artificial Intelligence. bioRxiv.

[315] Jumper J., Evans R., Pritzel A., Green T., Figurnov M., Ronneberger O., Tunyasuvunakool K., Bates R., Žídek A., Potapenko A. (2021). Highly Accurate Protein Structure Prediction with AlphaFold. Nature.

[316] Watson J.L., Juergens D., Bennett N.R., Trippe B.L., Yim J., Eisenach H.E., Ahern W., Borst A.J., Ragotte R.J., Milles L.F. (2023). De Novo Design of Protein Structure and Function with RFdiffusion. Nature.

[317] Mohan S., Kerry P.S., Bance N., Niikura M., Pinto B.M. (2014). Serendipitous Discovery of a Potent Influenza Virus A Neuraminidase Inhibitor. Angew. Chem. Int. Ed..

[318] Yuan M., Zhu X., He W.T., Zhou P., Kaku C.I., Capozzola T., Zhu C.Y., Yu X., Liu H., Yu W. (2022). A Broad and Potent Neutralization Epitope in SARS-Related Coronaviruses. Proc. Natl. Acad. Sci. USA.

[319] Walls A.C., Fiala B., Schäfer A., Wrenn S., Pham M.N., Murphy M., Tse L.V., Shehata L., O’Connor M.A., Chen C. (2020). Elicitation of Potent Neutralizing Antibody Responses by Designed Protein Nanoparticle Vaccines for SARS-CoV-2. Cell.

[320] Brouwer P.J.M., Antanasijevic A., Ronk A.J., Müller-Kräuter H., Watanabe Y., Claireaux M., Perrett H.R., Bijl T.P.L., Grobben M., Umotoy J.C. (2022). Lassa Virus Glycoprotein Nanoparticles Elicit Neutralizing Antibody Responses and Protection. Cell Host Microbe.

[321] Sesterhenn F., Yang C., Bonet J., Cramer J.T., Wen X., Wang Y., Chiang C.I., Abriata L.A., Kucharska I., Castoro G. (2020). De Novo Protein Design Enables the Precise Induction of RSV-Neutralizing Antibodies. Science.

[322] Mousa J.J., Sauer M.F., Sevy A.M., Finn J.A., Bates J.T., Alvarado G., King H.G., Loerinc L.B., Fong R.H., Doranz B.J. (2016). Structural Basis for Nonneutralizing Antibody Competition at Antigenic Site II of the Respiratory Syncytial Virus Fusion Protein. Proc. Natl. Acad. Sci. USA.

[323] Baum A., Fulton B.O., Wloga E., Copin R., Pascal K.E., Russo V., Giordano S., Lanza K., Negron N., Ni M. (2020). Antibody Cocktail to SARS-CoV-2 Spike Protein Prevents Rapid Mutational Escape Seen with Individual Antibodies. Science.

[324] Chen P., Behre G., Hebert C., Kumar P., Farmer MacPherson L., Graham-Clarke P.L., De La Torre I., Nichols R.M., Hufford M.M., Patel D.R. (2022). Bamlanivimab and Etesevimab Improve Symptoms and Associated Outcomes in Ambulatory Patients at Increased Risk for Severe Coronavirus Disease 2019: Results From the Placebo-Controlled Double-Blind Phase 3 BLAZE-1 Trial. Open Forum Infect. Dis..

[325] Dong J., Zost S.J., Greaney A.J., Starr T.N., Dingens A.S., Chen E.C., Chen R.E., Case J.B., Sutton R.E., Gilchuk P. (2021). Genetic and Structural Basis for SARS-CoV-2 Variant Neutralization by a Two-Antibody Cocktail. Nat. Microbiol..

[326] Focosi D., Casadevall A., Franchini M., Maggi F. (2024). Sotrovimab: A Review of Its Efficacy against SARS-CoV-2 Variants. Viruses.

[327] Vishweshwaraiah Y.L., Hnath B., Rackley B., Wang J., Gontu A., Chandler M., Afonin K.A., Kuchipudi S.V., Christensen N., Yennawar N.H. (2022). Adaptation-Proof SARS-CoV-2 Vaccine Design. Adv. Funct. Mater..

[328] Liu Q., Lu Y., Cai C., Huang Y., Zhou L., Guan Y., Fu S., Lin Y., Yan H., Zhang Z. (2024). A Broad Neutralizing Nanobody against SARS-CoV-2 Engineered from an Approved Drug. Cell Death Dis..

[329] World Health Organization (2024). Pathogens Prioritization: A Scientific Framework for Epidemic and Pandemic Research Preparedness.

[330] Bardhan M., Ray I., Roy S., Roy P., Thanneeru P., Twayana A.R., Prasad S., Bardhan M., Anand A. (2024). Disease X and COVID-19: Turning Lessons from India and the World into Policy Recommendations. Ann. Med. Surg..

